# Phox2a in Lateral Spinal Nucleus *Tac1*‐Positive Neurons Mediates Histamine‐Independent Acute Itch

**DOI:** 10.1111/cns.70639

**Published:** 2025-11-07

**Authors:** Yu‐Ling Chen, Zi‐Ang Li, Qing‐Zhen Wang, Xin‐Ran Wu, E. Mao, Yao‐Hua Liu, Zhi‐Ping Cai, Yun‐Qing Li, Zhen‐Zhen Kou

**Affiliations:** ^1^ Department of Human Anatomy and Histology and Embryology, School of Basic Medical Sciences The Fourth Military Medical University Xi'an China; ^2^ Department of Human Anatomy Baotou Medical College Baotou China; ^3^ Department of Human Anatomy, Histology and Embryology, School of Basic Medical Sciences Zhengzhou University Zhengzhou China; ^4^ Department of Anatomy, College of Basic Medicine Dali University Dali China

**Keywords:** chloroquine, histamine‐independent acute itch, lateral spinal nucleus, Phox2a, Tac1

## Abstract

**Aims:**

While acute itch comprises histamine‐dependent and ‐independent subtypes, critical mechanisms underlying histamine‐independent itch remain poorly understood. This study investigates the role of paired‐like homeobox 2a (Phox2a) in tachykinin 1‐positive (Tac1^+^) neurons of the lateral spinal nucleus (LSN) as a novel target for histamine‐independent pruritus intervention.

**Methods:**

We combined chemogenetic manipulation (viral‐mediated neuronal activation/inhibition), whole‐cell patch‐clamp recordings, immunohistochemistry, fluorescence in situ hybridization, Western blotting, and behavioral assays to investigate the role of LSN^Tac1^ neurons and Phox2a in itch modulation.

**Results:**

LSN^Tac1^ neurons were specifically activated during chloroquine (CQ)–induced histamine‐independent itch. Chemogenetic activation of these neurons exacerbated scratching, whereas inhibition suppressed itch behavior. Notably, Phox2a, expressed in LSN^Tac1^ neurons, was downregulated during CQ‐induced itch. Overexpression of Phox2a in LSN^Tac1^ neurons significantly alleviated CQ‐evoked scratching and was accompanied by a reduction in spontaneous excitatory postsynaptic currents (sEPSCs) amplitude without a change in sEPSCs frequency.

**Conclusions:**

Our findings identify Phox2a in LSN^Tac1^ neurons as a selective regulator of histamine‐independent acute itch through presynaptic excitability. This highlights Phox2a as a novel therapeutic target for histamine‐independent pruritus intervention.

## Graphical abstract

Phox2a expression in LSN^Tac1^ neurons is selectively reduced in chloroquine‐induced itch. Overexpression of Phox2a alleviates this itch by the amplitude of spontaneous excitatory postsynaptic currents, revealing a potential treatment target for histamine‐independent pruritus.

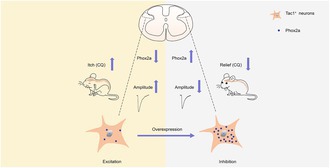

## 1 Introduction

Itch (pruritus) is an uncomfortable sensation that triggers the urge to scratch—a vital reflex for removing harmful stimuli from the skin [1]. However, excessive itch shifts from a protective response to a pathological condition, causing tissue damage, psychological distress, and substantial declines in patients' quality of life [2].

The lateral spinal nucleus (LSN), a critical constituent of the anterolateral system (ALS) within the central nervous system (CNS), is primarily located ventrolateral to the superficial laminae of the spinal dorsal horn (SDH) and extends rostrocaudally throughout the spinal cord [3, 4]. The LSN collaborates with neurons in laminae I and IV–V of the spinal cord to form the ALS neurotransmission pathway [5]. Although the roles of laminae I and IV–V in itch transmission are well characterized, the contribution of the LSN to this process remains unclear [6]. Neurons within the LSN exhibit neurochemical diversity, with particular attention given to tachykinin 1 (*Tac1*)‐expressing neurons, which encode substance P (SP), a neuropeptide implicated in itch signal transduction [7]. The contribution of the LSN^Tac1^ neurons to the modulation of itch processing requires additional investigation.

Recent studies have identified the transcription factor Paired‐like homeobox 2a (Phox2a) as a critical regulator of the LSN neurons [8]. Genetic analyses have further demonstrated that Phox2a‐expressing neurons represent the predominant neuronal population in the LSN neurons [9]. Previous studies have demonstrated that a substantial population of Phox2a‐expressing cells in the SDH co‐express *Tac1* mRNA [10, 11]. Notably, loss of Phox2a in the spinal cord leads to reduced innervation of target ALS neurons [9]. The specific involvement of Phox2a in the LSN^Tac1^ neurons for itch signal processing through spinal projection neurons requires further investigation.

Based on these considerations, we hypothesized that Phox2a in the LSN^Tac1^ neurons participates in acute itch. In the present study, approaches were applied to investigate the role of Phox2a in the LSN^Tac1^ neurons in itch neurotransmission. Our findings aimed to identify novel therapeutic targets and provide insights for the development of novel itch treatment strategies.

## 2 Materials and Methods

### 2.1 Animals

A total of 65 adult C57BL/6J mice (purchased from the Laboratory Animal Center of the Fourth Military Medical University), 48 adult Tac1‐Cre mice (RRID: IMSR_JAX: 021877), 2 adult vGluT2‐Cre mice (vesicular glutamate transporter 2‐Cre; RRID: IMSR_JAX: 028863), and 4 adult Ai9‐TdTomato mice (RRID: IMSR_JAX: 007909) (all purchased from The Jackson Laboratory, Bar Harbor, Maine, USA, except C57BL/6J; aged 8–12 weeks, weighing 20–30 g) were utilized in the present experiment. Because investigating sex differences was not the primary focus of our study, female mice were excluded from the experiments [12]. All animals were housed in a standard 12 h light/dark cycle environment with food and water provided *ad libitum*. The ambient temperature was maintained at 22°C–26°C, and the humidity was controlled within the range of 40%–70%. All experimental designs were optimized to minimize the number of animals used and reduce animal distress. All experimental procedures were approved by the Animal Care and Use Committee at The Fourth Military Medical University (NO: IACUC‐20210356, Xi'an, China).

### 2.2 Stereotaxic Injection

All mice were handled for theee consecutive days prior to stereotactic injection procedures to minimize stress. Mice were fixed on a stereotaxic apparatus (RWD Life Science, Shenzhen, China) after being deeply anesthetized with an intraperitoneal (*i.p*.) injection of pentobarbital sodium (40 mg/kg).

The injection coordinates are listed below: LSN medial‐lateral (ML), ±0.8 mm; and dorsal‐ventral (DV), ±0.2 mm. All viruses were injected at a rate of 10 nL/min and kept at the injection sites for 10 min before being withdrawn to avoid leakage. All viruses were purchased from BrainVTA Co. Ltd. (Wuhan, China).

For the chemogenetic manipulation of the LSN neurons, we administered injections of the following viral constructs: rAAV2/9‐hSyn‐hM3Dq‐eGFP‐WPRE‐hGH pA (100 nL, PT‐0152), rAAV2/9‐hSyn‐hM4Di‐eGFP‐WPRE‐hGH pA (100 nL, PT‐0153), or the control rAAV2/9‐hSyn‐eGFP‐WPRE‐hGH pA (100 nL, PT‐1990) into the bilateral LSN of C57BL/6J mice to manipulate the LSN neurons.

For the chemogenetic manipulation of the LSN^Tac1^ neurons, we administered injections of the following viral constructs: the rAAV2/9‐EF1α‐DIO‐hM3Dq‐eYFP‐WPRE‐hGH pA (100 nL, PT‐0816), rAAV2/9‐EF1α‐DIO‐hM4Di‐eYFP‐WPRE‐hGH pA (100 nL, PT‐0815), or the control rAAV2/9‐EF1α‐DIO‐eYFP‐WPRE‐hGH pA (100 nL, PT‐0012) into the bilateral LSN of Tac1‐Cre mice to specifically manipulate the LSN^Tac1^ neurons.

For overexpression of the Phox2a in the LSN neurons, we administered injections of the following viral constructs: the rAAV2/8‐hSyn‐Cre‐WPRE‐hGH pA (50 nL, PT‐0136) was mixed with the rAAV2/8‐EF1α‐DIO‐Phox2a‐2A‐mCherry‐ WPRE pA (50 nL, PT‐10670) or the control rAAV2/8‐EF1α‐DIO‐mCherry‐WPRE pA (50 nL, PT‐0013) in a 1:1 ratio and injected into the bilateral LSN of C57BL/6J mice.

For overexpression of Phox2a in the LSN^Tac1^ neurons, we administered injections of the following viral constructs: the rAAV2/8‐EF1α‐DIO‐Phox2a‐2A‐mCherry‐WPRE pA (100 nL, PT‐0153) or the control rAAV2/8‐EF1α‐DIO‐mCherry‐WPRE pA (100 nL, PT‐3325) into the bilateral LSN of Tac1‐Cre mice.

### 2.3 Cervical Cord Slice Preparation

All procedures were as previously described [13]. The mice were anesthetized and perfused transcardially with 50 mL of 0.01 M phosphate‐buffered saline (PBS). Subsequently, mice were perfused with 150 mL of 4% (*w/v*) paraformaldehyde in 0.1 M phosphate buffer. After perfusion, the spinal cord was removed. Then, the spinal cord was immersed in 30% (*w/v*) sucrose. Next, the spinal cord was cut into 30 μm transverse section slices. Sections were divided into six sets. Sections from the first set of a six‐well plate were confirmed for the accuracy of the injection site. Sections from the second set were stained with Nissl staining to characterize the region of the LSN.

### 2.4 Immunohistochemical Staining

The experimental protocols were implemented as reported [14]. Sections from the third set were selected for c‐FOS immunohistochemical staining using the 3,3′‐diaminobenzidine (DAB) method. Sections were washed three times in 0.01 M PBS, followed by blocking in a blocking solution (donkey serum: 0.01 M PBS = 1:9) for 30 min at room temperature (RT). Then the following antisera were incubated sequentially: mouse anti‐c‐FOS (1:500, AB11959, Abcam, Cambridge, MA, USA) for 24 h at RT, and biotin‐donkey anti‐mouse IgG antibody (1:500, AP192B, Merck Millipore, CA, USA) for 4 h at RT. After three washes with 0.01 M PBS, the sections were stained using a solution containing 0.04% DAB (D12384, Sigma, MO, USA) and 0.003% hydrogen peroxide (H_2_O_2_) in a 0.05 M Tris–HCl buffer (pH 7.6). The tissue sections were mounted onto gelatin‐coated glass slides, dehydrated through a series of ascending ethanol concentrations, clarified, and covered with coverslips before being examined under light microscopy (AHBT3, Olympus, Tokyo, Japan).

The Tac1‐Ai9 mice were generated by crossing Tac1‐Cre mice with Ai9‐tdTomato reporter mice. In Tac1‐Ai9 mice, the tdTomato sequence is inserted into the *Tac1* gene locus of *Tac1* neurons, enabling *Tac1* neurons to exhibit red fluorescence [13]. Chloroquine (CQ, #C6628) or histamine (His, #H7250) was injected into the midline of the nape of Tac1‐Ai9 mice to induce c‐FOS expression in neurons. The mice were then placed in their original cage for 2 h before perfusion. Sections from the fourth set of the six‐well plate were selected for immunohistochemical staining. The primary antibody was mouse anti‐c‐FOS (1:500). The secondary antibody was Alexa 488 donkey anti‐mouse (1:500, A21202, Invitrogen, CA, USA).

Immunostaining methods were used to evaluate the double‐labeling of Phox2a/NeuN in the LSN of C57BL/6J mice. The primary antibodies used were rabbit anti‐Phox2a (1:200, #PA5‐34381, Invitrogen) and mouse anti‐NeuN (1:500, MAB377, Merck Millipore). The secondary antibodies used were Alexa 488 donkey anti‐rabbit (1:500, #A21206, Invitrogen) and Alexa 594 donkey anti‐mouse (1:500, #A21203, Invitrogen).

Immunostaining methods were used to evaluate the double‐labeling of Phox2a/tdTomato in the LSN of Tac1‐Ai9 mice. The primary antibody used was rabbit anti‐Phox2a (1:200). The secondary antibody used was Alexa 488 donkey anti‐rabbit (1:500).

Immunostaining was performed to evaluate the double‐labeling of Biocytin/mCherry in the LSN of Tac1‐Cre mice that received either rAAV2/8‐DIO‐Phox2a (experimental group) or rAAV2/8‐DIO‐mCherry (control group) injections. The antibody used was Alexa 488‐ avidin (1:400, #S32356, Invitrogen).

### 2.5 Fluorescence In Situ Hybridization

Fluorescence in situ hybridization (FISH) staining was performed as described in our report [15]. In this study, vGluT2‐Ai9 mice were stained for *Tac1* mRNA expression using FISH. Briefly, the Fluorescent RNA Labeling Kit (Roche Diagnostics, Basel, Switzerland) was employed to synthesize fluorescence‐labeled antisense single‐stranded RNA probes. Throughout the experiment, attention was paid to no enzyme manipulation, and all experimental equipment was autoclaved. All the phosphate buffer (PB) and PBS solutions used for fixation and cryoprotection were incubated with 0.1% (*v/v*) diethyl pyrocarbonate (DEPC, DH098‐2, Genview, FL, USA) overnight. Sections were first incubated in 0.1 M DEPC‐PB in 2% H_2_O_2_ and washed in 0.1 M DEPC‐PB for 10 min, 0.3% Triton X‐100 for 20 min, and acetylation solution for 20 min. After rinsing with 0.1 M DEPC‐PB, the sections were subjected to a prehybridization step for 1.5 h in a prehybridization buffer, which consisted of 5 × saline sodium citrate (SSC, 1 × SSC = 0.15 M NaCl and 0.015 M sodium citrate, pH 7.0), 2% (*w/v*) blocking reagent, 50% (*v/v*) formamide, 0.1% (*w/v*) N‐lauroylsarcosine (NLS), and 0.1% (*w/v*) sodium dodecyl sulfate (SDS). *Tac1* riboprobes were subsequently incorporated into the prehybridization buffer, achieving a final concentration of 1 μg/mL, and allowed to react under conditions of 56°C for a duration of 24 h. After two × 20 min washes in wash buffer at 58°C, the sections were incubated with 20 μg/mL ribonuclease A (RNase A) at 37°C for 30 min, followed by two × 20 min rinses in 2 × SSC and 0.2 × SSC, respectively. Subsequently, the signal amplification of *Tac1* mRNA was achieved using the biotinylated tyramine (BT) and glucose oxidase (GO) method. Sections were incubated for 30 min in a solution containing 3 μg/mL GO, 1.25 μM BT, 1% bovine serum albumin (BSA), and 2 mg/mL β‐D‐glucose in 0.1 M phosphate buffer, along with peroxidase‐conjugated anti‐digoxigenin sheep antibody, rabbit anti‐vGluT2 antibody, and mouse anti‐NeuN antibody. Sections were incubated with FITC‐Avidin (1:500, A2001, Vectorlabs, Burlingame, CA, USA), Alexa 594‐donkey anti‐rabbit (1:500, #A21207, Invitrogen), and Alexa 647‐donkey anti‐mouse (1:500, #A31571, Invitrogen) for 4 h in TBS (0.1 M of Tris–HCl buffered 0.9% saline).

### 2.6 Application of Agents

To test whether the LSN neurons were involved in the processing of acute itch and pain sensations, 20 C57BL/6J mice were randomly divided into 4 subgroups: the saline group, the CQ‐induced itch group, the His‐induced itch group, and the capsaicin‐induced pain group (*n* = 5 per group). Mice were briefly anesthetized with isoflurane (RWD Life Science Co., Guangzhou, China), and 20 μL of CQ (200 μg/20 μL, Sigma), His (200 μg/20 μL, Sigma), or capsaicin (1.6 μg/20 μL, Sigma) dissolved in normal saline were injected into the midline of the nape of each mouse intradermally (i.d.) using an insulin syringe (BD, Franklin Lakes, NJ, USA). Upon completion of the injection, the mice were placed into the behavioral acquisition device and recorded with a digital camera for 30 min to confirm the efficacy of the agents injected into mice. The mice were sacrificed 2 h post‐injection, and the LSN sections were subjected to c‐FOS immunohistochemical staining.

### 2.7 Whole‐Cell Patch‐Clamp Recordings

The experimental procedures were based on previous reports [16, 17]. Briefly, mice were decapitated after being anesthetized with 3% isoflurane, and the whole C1–C8 segment was quickly isolated and then submerged in ice‐cold (4°C) oxygenated (95% O_2_ and 5% CO_2_) artificial cerebrospinal fluid (ACSF), which consisted of (in mM): 124 NaCl, 25 NaHCO_3_, 2.5 KCl, 1 NaH_2_PO_4_, 2 CaCl_2_, 1 MgSO_4_, and 10 glucose. Transverse slices (300 μm thick) containing the LSN were cut using a vibrating microtome (Leica VT 1200s, Heidelberger, Nussloch, Germany). The recordings were conducted in either voltage‐clamp or current‐clamp mode utilizing an Axon 700B amplifier (Molecular Devices, San Jose, CA, USA).

To systematically evaluate the excitability of the LSN^Tac1^ neurons under diverse experimental conditions, we employed three approaches: First, electrophysiological properties were characterized in mice under three conditions: CQ‐induced His‐independent itch, His‐dependent itch and saline control. Subsequently, to investigate Phox2a overexpression effects, we compared the LSN^Tac1^ neurons with either rAAV2/8‐DIO‐Phox2a overexpression (experimental) or rAAV2/8‐DIO‐mCherry (control) under non‐stimulated conditions. Finally, we examined the combined effects by analyzing Phox2a‐overexpressing versus control virus‐expressing neurons across all models (CQ/His/saline).

Electrophysiological recordings were performed using both voltage‐clamp and current‐clamp configurations, depending on the experimental protocol. For sEPSC recordings, cells were held at −70 mV in voltage‐clamp mode for 3 min. sEPSCs were identified and analyzed using MiniAnalysis software (Synaptosoft Inc., Decatur, GA, USA) based on two primary criteria: a fixed amplitude threshold set above the baseline noise level (typically > 5 pA), and characteristic waveform kinetics featuring a rapid rising phase followed by a slower exponential decay; only events that unequivocally exceeded the noise level and exhibited this classic synaptic current morphology were included in the analysis, with all detection parameters applied consistently across all recorded neurons.

The rheobase is the minimum current strength capable of eliciting at least one action potential (AP). For excitability measurements, current‐clamp mode was used, in which supra‐threshold depolarizing currents ranging from 0 to 100 pA (30 ms duration) were delivered in increments of 5 pA to assess firing properties. The amplitude of the AP was determined by measuring the distance between the threshold's equipotential point and the peak of the spike. An alternative protocol was employed, involving the application of depolarizing currents ranging from 0 to 100 pA (400 ms duration) in steps of 5 pA, to investigate alterations in the spike count.

### 2.8 Chemogenetic Manipulation

In the chemogenetic manipulation, all behavioral tests were carried out 40 min after an intraperitoneal (i.p.) injection of 0.1 mg/kg clozapine (C6305, Sigma) and completed within 6 h. The clozapine was dissolved in 0.9% saline. Saline injection was administered to the control group. A 3‐day interval was observed between different behavioral experiments.

### 2.9 Itch Behavioral Tests

Mice were placed in a (15 × 10 × 25 cm^3^) rectangular opaque box for 30 min over three consecutive days of acclimation [13]. Subsequently, 0.9% saline (20 μL), CQ (200 μg/20 μL), or His (200 μg/20 μL) was i.d. injected into the midline of the nape. Scratching behaviors were recorded for 30 min after injection, with counting initiated by the first scratch from either hind paw directed toward the midline region of the nape.

### 2.10 Open Field Test (OFT)

The OFT was employed to assess the locomotor abilities and anxiety‐related behaviors in mice [18]. Mice were placed into the OFT room 30 min before the tests to adapt to the environment. At the beginning of the experiment, mice were placed into the central area (50 × 50 × 40 cm^3^), and their movement was recorded for 15 min. Subsequently, the apparatus was sanitized with 75% ethyl alcohol before testing the next mouse. After the test, data were analyzed using an automated infrared detection system.

### 2.11 Elevated Plus Maze (EPM)

The mouse was placed in the EPM chamber 30 min in advance to adapt to the environment [18]. The EPM apparatus comprises two opposing open arms (30 × 5 cm), two opposing closed arms (30 × 5 cm), and a central zone (5 × 5 cm). The platform was positioned at a height of 50 cm above the floor level. Typically, the mice were individually placed in the central area of the maze and allowed 5 min of free exploration. The data were measured with an automated infrared detection system.

### 2.12 Western Blotting

The LSN of the C1‐C8 segment was removed after mice were decapitated and placed in lysis buffer containing protease inhibitors and phosphatase inhibitors (Roche, Switzerland) [19]. Protein concentration was measured using a bicinchoninic acid (BCA) assay (Pierce, Rockford, IL, USA). Equal amounts of protein samples were electrophoresed by SDS‐PAGE on 15% polyacrylamide gel. After electrophoretic transfer to PVDF membrane (Millipore, Bedford, MA, USA), the blots were blocked with a blocking buffer (5% nonfat dry milk in TBS‐T) for 2 h at RT and then incubated with primary antibodies overnight at 4°C: rabbit anti‐Phox2a antibody (1:1000). After incubation of the membrane with peroxidase‐conjugated anti‐rabbit secondary antibodies (Santa Cruz Biotechnology, Dallas, TX, USA) for 2 h at RT, the reaction products were visualized using enhanced chemiluminescence (Amersham Life Science, Amersham, UK).

### 2.13 Statistical Analysis

GraphPad Prism 9.0 software (GraphPad, San Diego, CA, USA) was used for statistical analyses. Unpaired *t*‐test, Chi‐square test, One‐way ANOVA or Two‐way ANOVA were used to analyze the original data. Neural cell bodies in the LSN were counted on 20 sections from one well in the six‐well plate according to a previous report, and summarized as data for each mouse [4]. All c‐FOS‐positive neuron counts were quantified from standardized bilateral regions of interest (ROIs) using ImageJ software within the target LSN [20], with identical ROI positioning across all experimental groups (CQ‐, His‐, saline‐, and capsaicin‐treated). The area for each unilateral LSN ROI was fixed at 0.26 mm^2^ (260,00.00 μm^2^), resulting in a total quantified area of 0.52 mm^2^ per animal (bilaterally). Data are presented as mean ± SD *p* < 0.05 was considered statistically significant.

## 3 Results

### 3.1 Acute Itch Stimuli Increase the Expression of c‐FOS in the LSN

**FIGURE 1:**
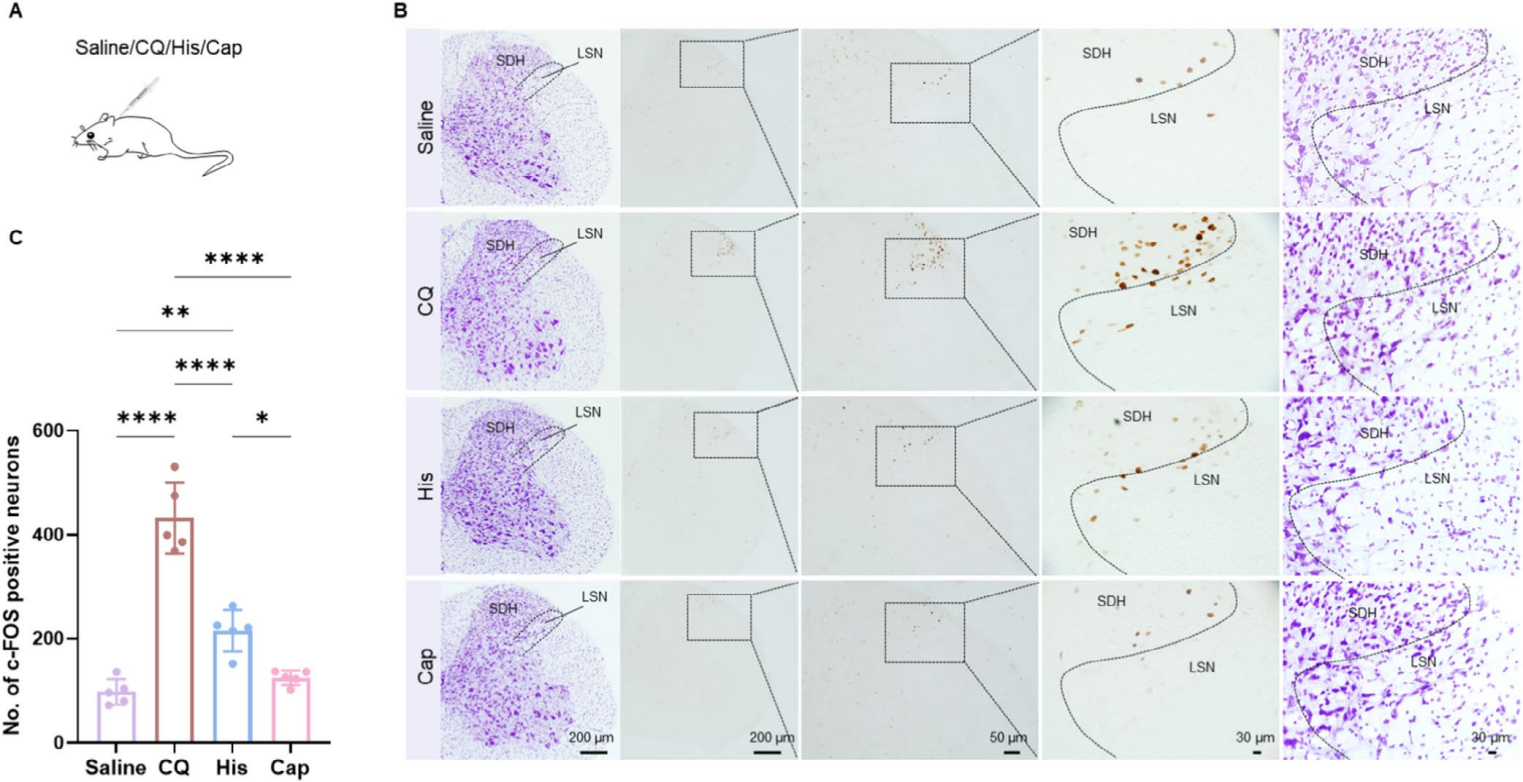
c‐FOS expression in the LSN induced by acute itch and acute pain stimulation. (A) Schematic diagram of subcutaneous injections in the nape of mice with saline, CQ, His, or capsaicin. (B) c‐FOS‐positive cells were observed in the LSN following intradermal injections of saline, CQ, His or Capsaicin, respectively, with Nissl staining of the adjacent section (purple). The columns (top to bottom): Saline → CQ → His → Cap, while rows (left to right): Nissl‐stained sections (10×) → DAB‐stained spinal cord (10×) → magnified DAB‐stained LSN regions (20×) → higher magnified DAB‐stained LSN regions (60×) → higher magnified Nissl‐stained LSN regions (60×). (C) The number of c‐FOS‐positive cells in itch and pain conditions (one‐way ANOVA, *F*_(3,16)_ = 64.51, *p* < 0.0001; Saline vs. CQ: *p* < 0.0001; Saline vs. His: *p* = 0.0023 < 0.01; CQ vs. Capsaicin: *p* < 0.0001; His vs. Capsaicin: *p* = 0.0178 < 0.05; *n* = 5 per group). p* < 0.05, **p < 0.01, ****p < 0.0001. Cap: capsaicin; CQ, chloroquine; His, histamine; LSN, lateral spinal nucleus; SDH, spinal dorsal horn.

After injection of CQ or His (Figure 1A), mice exhibited robust scratching behaviors (CQ: 181.40 ± 28.77 bouts; His: 60.20 ± 17.11 bouts), confirming successful establishment of acute itch models. In contrast, saline and capsaicin induced significantly fewer scratches (Saline: 9.20 ± 9.76 bouts; Capsaicin: 34.80 ± 7.85 bouts, Table 1, Figure S1A).

**TABLE 1:** The number of scratching instances induced by different agents in C57BL/6J mice (*n* = 5).

	Saline	CQ	His	Capsaicin
Mouse 1	15	174	58	41
Mouse 2	1	163	41	26
Mouse 3	7	206	74	44
Mouse 4	23	216	81	35
Mouse 5	0	148	47	28
Mean ± SD	9.20 ± 9.76	181.40 ± 28.77****	60.20 ± 17.11***	34.80 ± 7.85

*Note:* Data are presented as the raw counts for each individual mouse. The group mean ± S.D. is also shown. ***p < 0.001, ****p < 0.0001 vs. saline group (one‐way ANOVA with Dunnett's post hoc test).

Abbreviations: CQ, chloroquine; His, histamine.

Study has shown that c‐FOS proteins are widely recognized and used as markers for neuronal activity [21]. We observed a statistically significant increase in c‐FOS‐expressing neurons in the LSN following CQ or His injection compared with saline and capsaicin controls (Figure 1B,C). The number of c‐FOS‐positive neurons was 432.00 ± 68.60 (CQ), 215.2 ± 40.03 (His), 97.60 ± 24.92 (saline), and 124.80 ± 14.13 (capsaicin) respectively (Table 2). Notably, the CQ‐treated group exhibited the most pronounced increase in the number of c‐FOS‐expressing neurons in the LSN. Importantly, a strong positive correlation was observed between the number of scratching bouts and the count of c‐FOS‐positive neurons (*R* ^2^ = 0.98, *p* < 0.0001, Figure S1B).

**TABLE 2:** Number of c‐FOS in the bilateral side of the LSN in C57BL/6J mice (*n* = 5).

	Saline	CQ	His	Capsaicin
Mouse 1	104	399	221	136
Mouse 2	80	386	152	102
Mouse 3	96	475	225	137
Mouse 4	136	531	263	126
Mouse 5	72	369	215	123
Total	488	2160	1076	624
Mean ± SD	97.60 ± 24.92	432.00 ± 68.60	215.20 ± 40.03	124.80 ± 14.13

*Note:* Cell counts were performed on 20 sections of 30 μm thickness (five mice in each model). These data were expressed as the mean ± SD.

Abbreviations: CQ, chloroquine; His, histamine.

The results imply that the LSN neurons are activated in the acute itch model and are mainly associated with CQ‐induced itch.

### 3.2 Chemogenetic Activation/Inhibition of the LSN Increases/Decreases Acute Itch‐Induced Scratching Behaviors

**FIGURE 2:**
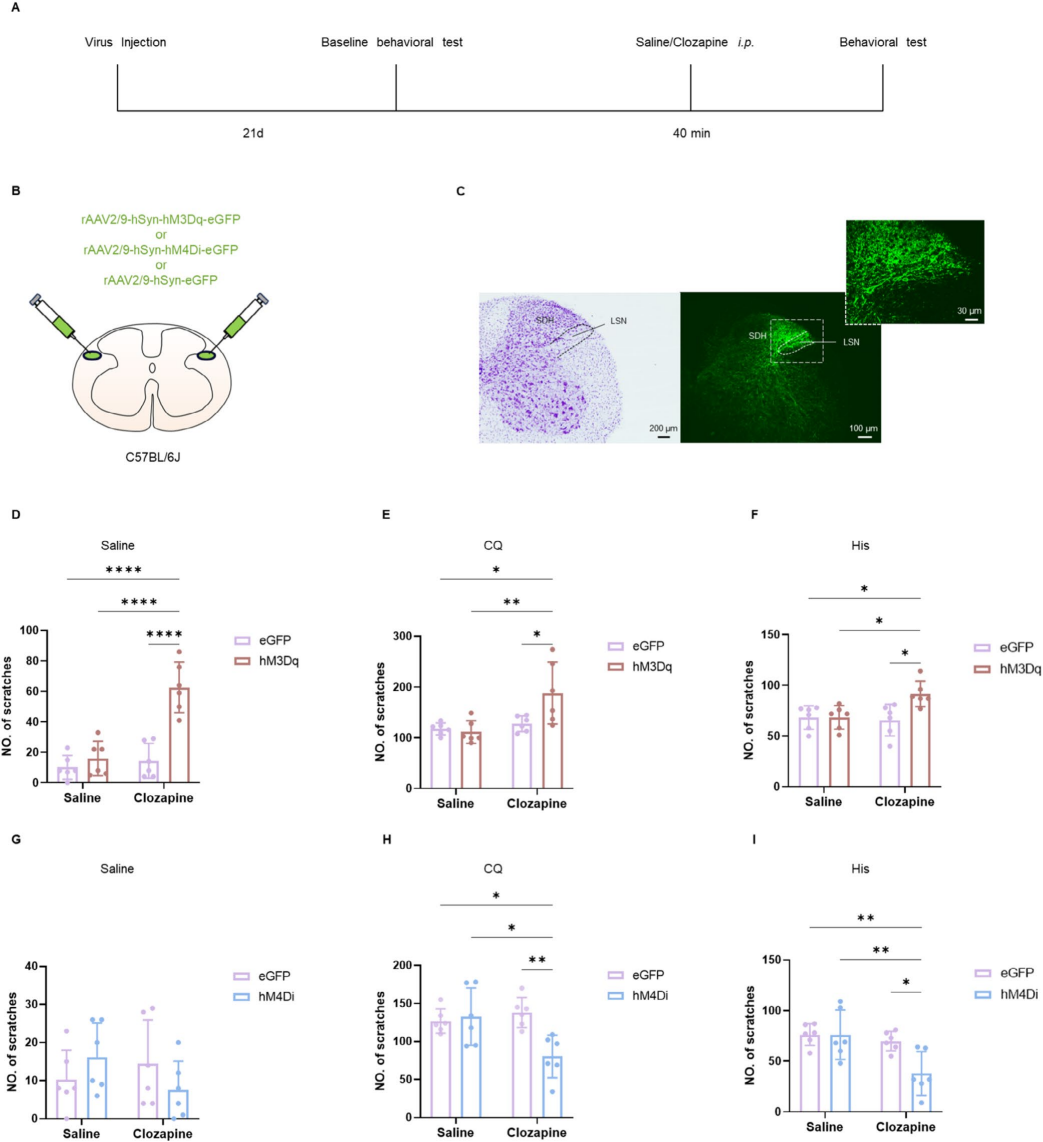
Chemogenetic modulation of the LSN neurons in itch. (A) Viral strategy and the timeline of the behavioral experiment. (B) Schematic plot showing injection of hM3Dq/hM4Di/eGFP virus into the bilateral side LSN. (C) The injection site of the virus in the LSN (middle) with a Nissl staining of the adjacent section (left). The corresponding images of the rectangles in (middle) were enlarged in (right). (D–F) Chemogenetic activation of the LSN promoted scratching behaviors induced by saline (two‐way ANOVA, interaction *F*_(1,20)_ = 18.04, *p* = 0.0004; eGFP‐Saline vs. hM3Dq‐Clozapine: *p* < 0.0001; hM3Dq‐Saline vs. hM3Dq‐Clozapine: *p* < 0.0001; eGFP‐Clozapine vs. hM3Dq‐Clozapine: *p* < 0.0001, *n* = 6 per group), CQ (two‐way ANOVA, interaction *F*_(1,20)_ = 5.787, *p* = 0.0259; eGFP‐Saline vs. hM3Dq‐Clozapine: *p* = 0.0105 < 0.05; hM3Dq‐Saline vs. hM3Dq‐Clozapine: *p* = 0.0051 < 0.01; eGFP‐Clozapine vs. hM3Dq‐Clozapine: *p* = 0.0344 < 0.05, *n* = 6 per group) and His (two‐way ANOVA, interaction *F*_(1,20)_ = 5.884, *p* = 0.0249; eGFP‐Saline vs. hM3Dq‐Clozapine: *p* = 0.0256 < 0.05; hM3Dq‐Saline vs. hM3Dq‐Clozapine: *p* = 0.0268 < 0.05; eGFP‐Clozapine vs. hM3Dq‐Clozapine: *p* = 0.0124 < 0.05, *n* = 6 per group). (G–I) Inhibitory chemogenetic manipulation of the LSN neurons displayed no effect on saline‐induced scratching behaviors (two‐way ANOVA, interaction *F*_(1,20)_ = 3.064, *p* = 0.0954; eGFP‐Saline vs. hM4Di‐Clozapine: *p* = 0.9969; hM4Di‐Saline vs. hM4Di‐Clozapine: *p* = 0.5179; eGFP‐Clozapine vs. hM4Di‐Clozapine: *p* = 0.7330, *n* = 6 per group), whereas attenuated CQ‐induced (two‐way ANOVA, interaction *F*_(1,20)_ = 8.494, *p* = 0.0086; eGFP‐Saline vs. hM4Di‐Clozapine: *p* = 0.0068 < 0.01; hM4Di‐Saline vs. hM4Di‐Clozapine: *p* = 0.0068 < 0.01; eGFP‐Clozapine vs. hM4Di‐Clozapine: *p* = 0.0265 < 0.05, *n* = 6 per group) and His‐induced (two‐way ANOVA, interaction *F*_(1,20)_ = 4.811, *p* = 0.0403; eGFP‐Saline vs. hM4Di‐Clozapine: *p* = 0.0322 < 0.05; hM4Di‐Saline vs. hM4Di‐Clozapine: *p* = 0.0143 < 0.05; eGFP‐Clozapine vs. hM4Di‐Clozapine: *p* = 0.0065 < 0.001, *n* = 6 per group) scratching behaviors when applied with clozapine. *p < 0.05, **p < 0.01, ****p < 0.0001. LSN, lateral spinal nucleus; SDH, spinal dorsal horn.

The morphological results indicate that the LSN neurons are activated by acute itch. To further investigate the role of the LSN neurons in acute itch, we employed the Designer Receptors Exclusively Activated by Designer Drugs (DREADD) system, which allows for the expression of genetically engineered muscarinic receptors to modulate neural activity in the LSN. As illustrated in Figure 2A, 3 weeks after the virus injection, the C57BL/6J mice received an i.p. injection of clozapine or saline, followed by an *i.d*. saline injection into the nape. Specifically, rAAV2/9‐ hSyn‐hM3Dq‐eGFP (activation group) and the control virus rAAV2/9‐hSyn‐eGFP (control group) were injected into the bilateral LSN of C57BL/6J mice, respectively (Figure 2B,C). Chemogenetic activation of the LSN neurons significantly increased spontaneous scratching behaviors in hM3Dq‐injected groups compared with eGFP controls (Figure 2D). Furthermore, this neuronal activation also amplified CQ‐induced scratching behaviors relative to saline‐treated mice (Figure 2E). This potentiating effect was observed in His‐treated groups as well (Figure 2F). Notably, the chemogenetic activation of the LSN neurons did not affect locomotor abilities in the OFT (Figure S2A–E) or anxiety‐like behaviors in the EPM (Figure S2F–H). The above results indicate that the activation of the LSN neurons enhances acute itch‐induced scratching behaviors but does not participate in the anxiety‐like behaviors.

To examine the effects of inhibiting the LSN neurons in C57BL/6J mice, we stereotactically injected rAAV2/9‐hSyn‐hM4Di‐eGFP (inhibition group) and the control virus rAAV2/9‐hSyn‐eGFP (control group) into the bilateral LSN of C57BL/6J mice, respectively. In acute itch models, administration of clozapine to the hM4Di‐injected group significantly reduced the number of scratches induced by either His or CQ, compared with the saline‐treated control group (Figure 2G–I). The chemogenetic inhibition of the LSN neurons did not affect locomotor abilities in the OFT (Figure S3A–E) or anxiety‐like behaviors in the EPM (Figure S3F–H). These findings demonstrate that inhibiting the LSN neurons alleviates acute itch‐induced scratching behaviors in mice.

Our data demonstrate that the LSN neurons are involved in itch neurotransmission, primarily by facilitating the propagation of pruriceptive information.

### 3.3 Chemogenetic Activation/Inhibition of the LSN^Tac1^ Neurons Increases/Decreases Acute Itch‐Induced Scratching Behaviors

**FIGURE 3:**
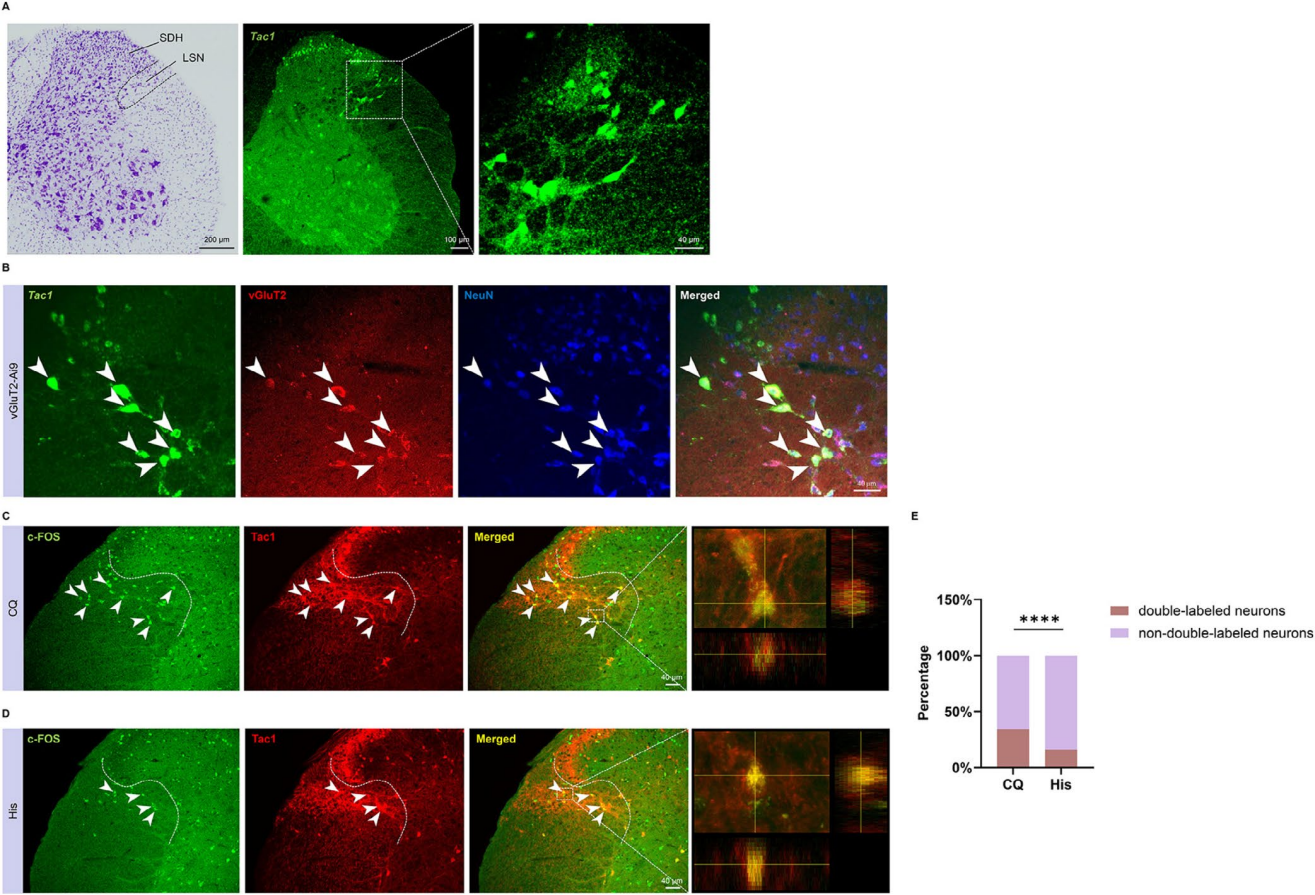
The Tac1/vGluT2 double‐labeled neurons in the LSN and c‐FOS expression in the LSN^Tac1^ neurons induced by acute itch stimulation. (A) The localization of the *Tac1*‐positive neurons (middle) with the Nissl staining of the adjacent section (left). The corresponding images of the rectangles were enlarged in (right). (B) The *Tac1*‐positive neurons (green), vGluT2‐labeled neurons (red) and NeuN‐immunoreactive neurons (blue) and the Tac1/vGluT2/NeuN triple‐labeled neurons (merged) were observed in the LSN. (C) Images of c‐FOS expressions (green) in the Tac1‐positive neurons (red) with intradermal injection of CQ in the nape of mice. The corresponding images of the rectangles were enlarged in (right). (D) Images of c‐FOS expressions (green) in the Tac1‐positive neurons (red) with intradermal injection of His in the nape of mice. The corresponding images of the rectangles were enlarged in (right). (E) Tac1/c‐FOS double‐labeled neurons in the LSN was significantly higher in CQ‐induced itch models compared to His‐induced itch (Chi‐square test: *χ* ^2^ = 321.1, df = 1, ****p < 0.0001; data from Tables 3 and 4). CQ, chloroquine; His, histamine; LSN, lateral spinal nucleus; SDH, spinal dorsal horn.

Combined FISH utilizing *Tac1* riboprobes with immunofluorescent staining, our results indicated the presence of *Tac1* mRNA within the LSN (Figure 3A). The vGluT2‐Ai9 mice, which allow for the insertion of the tdTomato sequence into the *vGluT2* gene locus of *vGluT2* neurons, enable *vGluT2* neurons to exhibit red fluorescence. We observed that in the LSN, the *Tac1*/vGluT2/NeuN triple‐labeled neurons demonstrate that approximately 90.49% ± 2.47% of *Tac1*‐positive neurons are vGluT2‐positive, as confirmed by in situ hybridization (Figure 3B, Table 3). This finding indicates that *Tac1*‐positive neurons in the LSN are primarily excitatory neurons.

**TABLE 3:** The number and percentages of Tac1/vGluT2/NeuN triple‐labeled neurons in the LSN (*n* = 5).

	Tac1	vGluT2	NeuN	Tac1 + vGluT2	Tac1/vGluT2 (%)	Tac1/NeuN (%)	Tac1 + vGluT2/Tac1 (%)
Mouse 1	728	816	865	656	89.22	84.16	90
Mouse 2	963	1230	1360	863	78.29	70.81	89.61
Mouse 3	866	929	1026	779	93.22	84.41	89.95
Mouse 4	698	781	801	661	89.37	87.14	84.70
Mouse 5	796	876	963	702	90.87	82.66	88.19
Total	4051	4632	5015	3661			
Mean ± SD	810.20 ± 107.25	926.40 ± 178.92	1003.00 ± 217.62	732.20 ± 88.16	88.19 ± 5.77	81.84 ± 6.37	90.49 ± 2.47

*Note:* Cell counts were performed on 20 sections of 30 μm thickness (five mice in each model). These data were expressed as the mean ± SD Tac1 + vGluT2, indicates the number of Tac1/vGluT2 double‐labeled neurons; Tac1/vGluT2 (%), indicates the percentage of Tac1‐labeled neurons to the total number of vGluT2‐labeled neurons; Tac1/NeuN (%), indicates the percentage of Tac1‐labeled neurons to the total number of NeuN‐labeled neurons; Tac1 + vGluT2/Tac1 (%), indicates the percentage of Tac1/vGluT2 double‐labeled neurons to the total number of Tac1‐labeled neurons.

We next investigated the involvement of the LSN^Tac1^ neurons in itch processing. In Tac1‐Ai9 mice, quantitative analyses revealed that 16.39% ± 6.78% of Tac1‐positive neurons showed c‐FOS/Tac1 double‐labeling following His‐induced itch (Figure 3D, Table 4), compared to 34.46% ± 5.18% in CQ‐induced acute itch models (Figure 3C, Table 5). The CQ group showed significantly higher LSN^Tac1^ neuronal activation than the His group (Figure 3E). These findings demonstrate that the LSN^Tac1^ neurons are more closely activated by CQ‐induced itch.

**TABLE 4:** The number and percentages of Tac1/c‐FOS double‐labeled neurons in the LSN (*n* = 5).

	Tac1	c‐FOS	Tac1 + c‐FOS	Tac1 + c‐FOS/Tac1 (%)
Mouse 1	719	201	108	15.02
Mouse 2	761	127	81	10.64
Mouse 3	934	213	95	10.17
Mouse 4	605	232	120	19.83
Mouse 5	810	334	213	26.30
Total	3829	1107	617	
Mean ± SD	765.80 ± 120.74	221.40 ± 74.48	123.40 ± 52.16	16.39 ± 6.78

*Note:* Cell counts were performed on 20 sections of 30 μm thickness (five mice in each model). These data were expressed as the mean ± SD Tac1 + c‐FOS, indicates the number of Tac1/c‐FOS double‐labeled neurons; Tac1 + c‐FOS/Tac1 (%), indicates the percentage of Tac1/c‐FOS double‐labeled neurons to the total number of Tac1‐labeled neurons.

**TABLE 5:** The number and percentages of Tac1/c‐FOS double‐labeled neurons in the LSN (*n* = 5).

	Tac1	c‐FOS	Tac1 + c‐FOS	Tac1 + c‐FOS/Tac1 (%)
Mouse 1	836	321	272	32.53
Mouse 2	720	301	246	34.17
Mouse 3	703	409	203	27.73
Mouse 4	532	313	191	35.90
Mouse 5	603	321	253	41.96
Total	3394	1665	1165	
Mean ± SD	678.80 ± 116.50	333.00 ± 43.27	233.00 ± 34.47	34.46 ± 5.18

*Note:* Cell counts were performed on 20 sections of 30 μm thickness (five mice in each model). These data were expressed as the mean ± SD Tac1 + c‐FOS, indicates the number of Tac1/c‐FOS double‐labeled neurons; Tac1 + c‐FOS/Tac1 (%), indicates the percentage of Tac1/c‐FOS double‐labeled neurons to the total number of Tac1‐labeled neurons.

**FIGURE 4:**
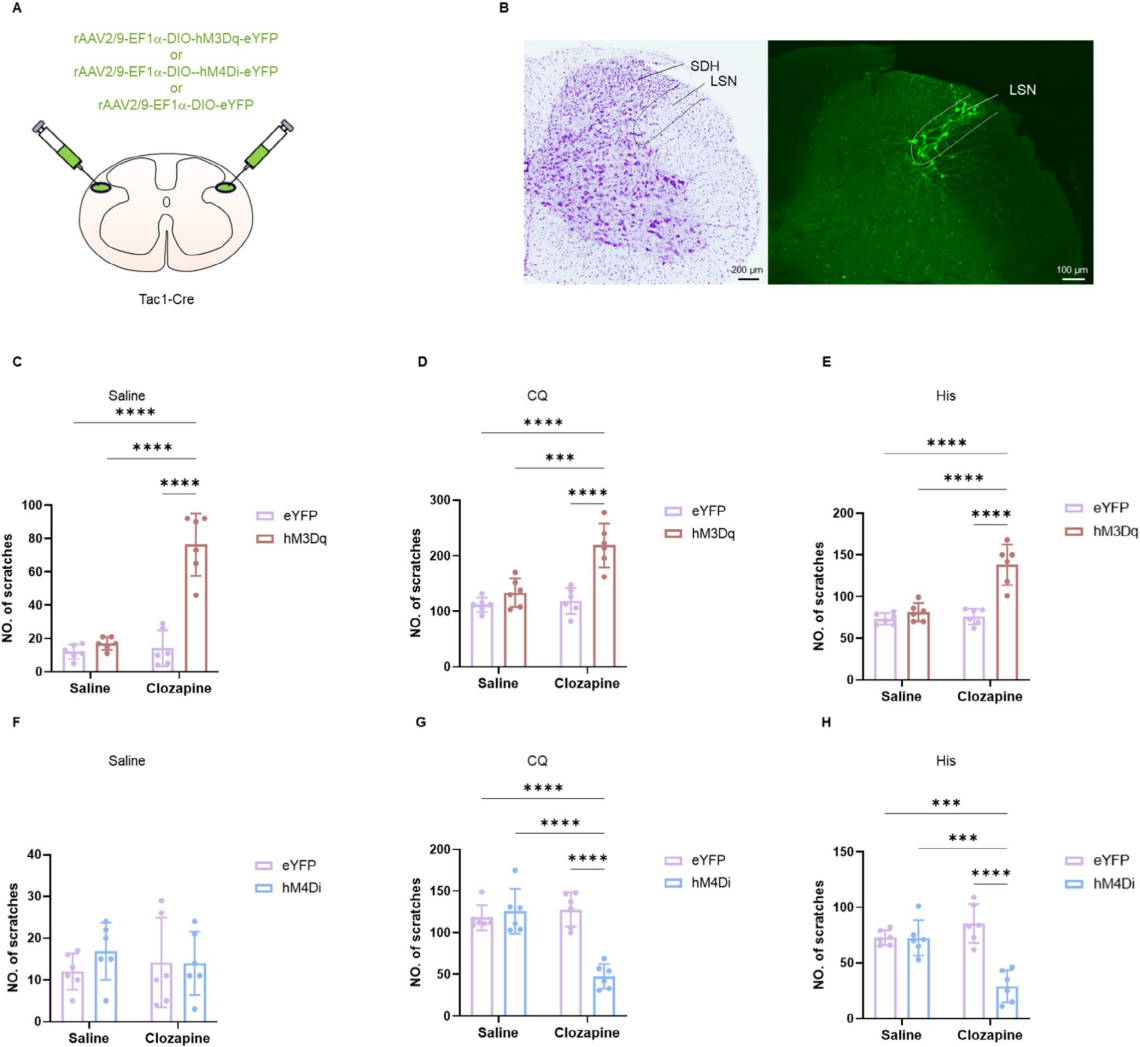
Chemogenetic modulation of the LSN^Tac1^ neurons in itch. (A) Schematic diagram showing the injection of the Cre recombinase‐dependent rAAV virus‐expressing DIO‐hM3Dq/hM4Di/eYFP into the bilateral side LSN of Tac1‐Cre mice. (B) The injection site of the virus in the LSN^Tac1^ neurons (right) with a Nissl staining of the adjacent section (left). (C–E) Chemogenetic activation of the LSN^Tac1^ neurons promoted scratching behaviors induced by saline (two‐way ANOVA, interaction *F*_(1,20)_ = 39.53, *p* < 0.0001; eYFP‐Saline vs. hM3Dq‐Clozapine: *p* < 0.0001; hM3Dq‐Saline vs. hM3Dq‐Clozapine: *p* < 0.0001; eYFP‐Clozapine vs. hM3Dq‐Clozapine: *p* < 0.0001, *n* = 6 per group), CQ (two‐way ANOVA, interaction *F*_(1,20)_ = 12.55, *p* = 0.0020; eYFP‐Saline vs. hM3Dq‐Clozapine: *p* < 0.0001; hM3Dq‐Saline vs. hM3Dq‐Clozapine: *p* = 0.0001 < 0.001; eYFP‐ Clozapine vs. hM3Dq‐Clozapine: *p* < 0.0001, *n* = 6 per group) and His (two‐way ANOVA, interaction *F*_(1,20)_ = 20.85, *p* = 0.0002; eYFP‐Saline vs. hM3Dq‐Clozapine: *p* < 0.0001; hM3Dq‐Saline vs. hM3Dq‐Clozapine: *p* < 0.0001; eYFP‐Clozapine vs. hM3Dq‐Clozapine: *p* < 0.0001, *n* = 6 per group). (F–H) Inhibitory chemogenetic manipulation of the LSN^Tac1^ neurons displayed no effect on saline‐induced scratching behaviors (two‐way ANOVA, interaction *F*_(1,20)_ = 0.6283, *p* = 0.4373; eYFP‐Saline vs. hM4Di‐Clozapine: *p* = 0.9692; hM4Di‐Saline vs. hM4Di‐Clozapine: *p* = 0.9194; eYFP‐ Clozapine vs. hM4Di‐Clozapine: *p* > 0.9999, *n* = 6 per group), whereas attenuated CQ‐induced (two‐way ANOVA, interaction *F*_(1,20)_ = 28.59, *p* < 0.0001; eYFP‐Saline vs. hM4Di‐Clozapine: *p* < 0.0001; hM4Di‐Saline vs. hM4Di‐Clozapine: *p* < 0.0001; eYFP‐Clozapine vs. hM4Di‐Clozapine: *p* < 0.0001, *n* = 6 per group) and His‐induced (two‐way ANOVA, interaction *F*_(1,20)_ = 23.06, *p* = 0.0001; eYFP‐Saline vs. hM4Di‐Clozapine: *p* = 0.0002 < 0.001; hM4Di‐Saline vs. hM4Di‐Clozapine: *p* = 0.0002 < 0.01; eYFP‐Clozapine vs. hM4Di‐Clozapine: *p* < 0.0001, *n* = 6 per group) scratching behaviors when applied with clozapine. ***p < 0.001, ****p < 0.0001. LSN, lateral spinal nucleus; SDH, spinal dorsal horn.

To further investigate the involvement of the LSN^Tac1^ neurons in itch neurotransmission, we performed bilateral injections of rAAV2/9‐EF1α‐DIO‐hM3Dq‐eYFP (activation group) and the control virus rAAV2/9‐EF1α‐DIO‐eYFP into the LSN of Tac1‐Cre mice, respectively (Figure 4A,B). The results demonstrated that the chemogenetic activation of the LSN^Tac1^ neurons led to a significant increase in scratching behaviors elicited by saline (Figure 4C), CQ (Figure 4D) and His (Figure 4E) compared with the control group. Additionally, the chemogenetic activation of the LSN^Tac1^ neurons did not affect locomotor abilities in the OFT or anxiety‐like behaviors in the EPM compared with the control group (Figure S4A–H). In summary, activating the LSN^Tac1^ neurons enhances acute itch‐induced scratching behaviors.

To investigate the effects of chemogenetic inhibition of the LSN^Tac1^ neurons in Tac1‐Cre mice, the rAAV2/9‐DIO‐hM4Di‐eYFP (inhibition group), and the control virus rAAV2/9‐DIO‐eYFP (control group) were injected into the bilateral LSN of Tac1‐Cre mice, respectively. Administration of clozapine resulted in a significant reduction of itch behaviors induced by both the CQ and His models (Figure 4F–H). Similarly, the mice showed no significant differences in the OFT and EPM (Figure S5A–H). In summary, inhibiting the LSN^Tac1^ neurons reduces acute itch‐induced scratching behaviors.

Collectively, the results demonstrate the involvement of the LSN^Tac1^neurons in itch neurotransmission.

### 3.4 Mechanisms Underlying the Discrepancy in his‐ and CQ‐Induced Activation of the LSN^Tac1^ Neurons

**FIGURE 5:**
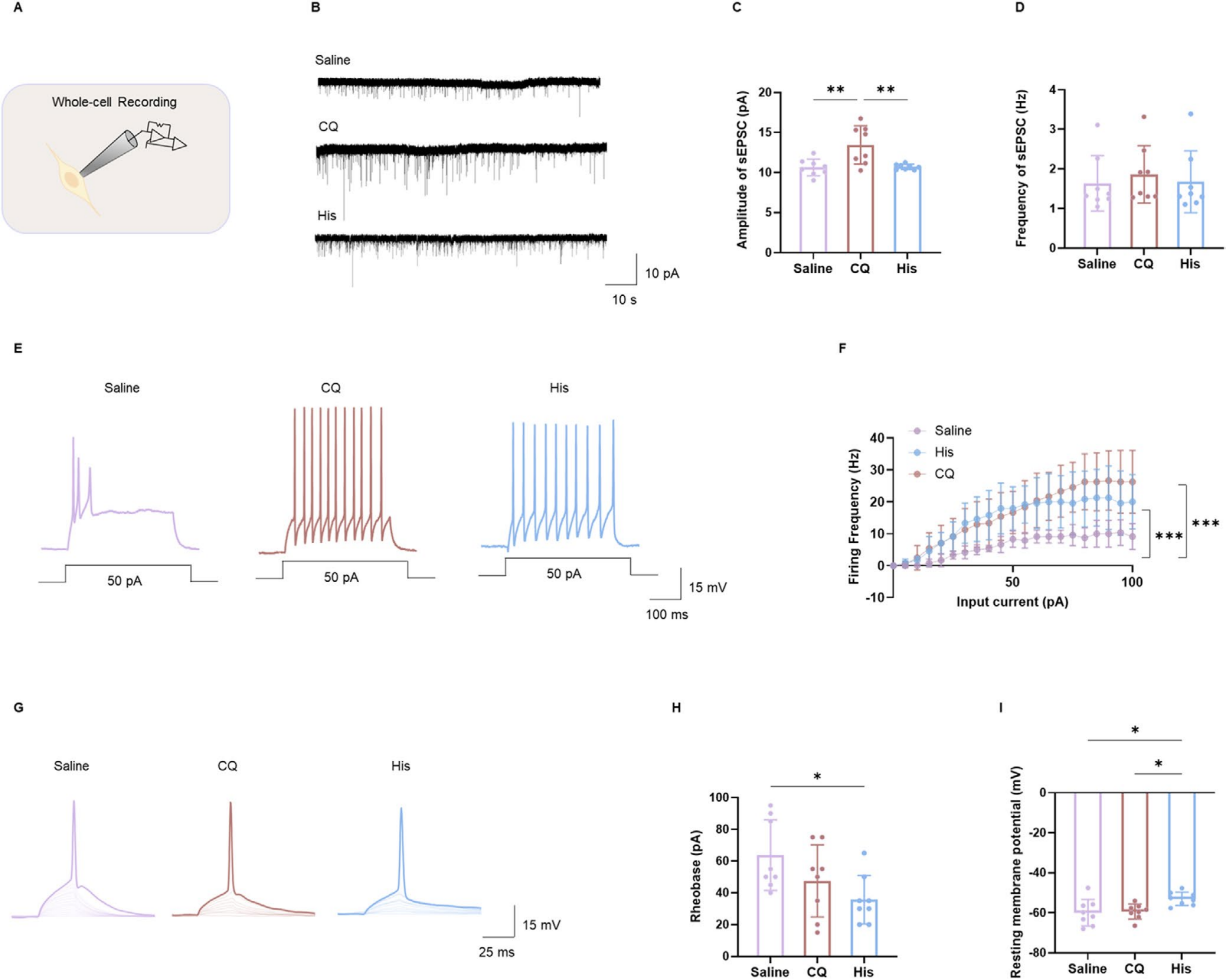
Neuronal excitability of the LSN^Tac1^ neurons in acute itch models. (A) Schematic diagram showing the recording of Tac1‐positive neurons (repeated for each successfully recorded neuron). (B) Sample traces of sEPSC recorded at −70 mV in neurons from the Saline, CQ and His groups. (C and D) Summary data of the amplitude and frequency of sEPSC (one‐way ANOVA, amplitude: Interaction *F*_(2,21)_ = 8.881, *p* = 0.0016; Saline vs. CQ: *p* = 0.0037 < 0.01; Saline vs. His: *p* = 0.9956; CQ vs. His: *p* = 0.0045 < 0.01; frequency: Interaction *F*_(2,21)_ = 0.2164, *p* = 0.8072; Saline vs. CQ: *p* = 0.8131; Saline vs. His: *p* = 0.9936; CQ vs. His: *P* = 0.8682; *n* = 8 neurons per group; mice = 3 per group). (E) Sample traces of action potential induced by depolarizing current (400 ms; 5 pA increments). (F) Summary data of the firing frequency induced by step‐wise depolarizing currents (two‐way ANOVA, interaction *F*_(40,315)_ = 1.465, *p* = 0.0404; Saline vs. CQ: *p* < 0.0001; Saline vs. His: *p* < 0.0001; CQ vs. His: *p* = 0.1662, *n* = 6 neurons per group, mice = 3 per group). (G) Sample traces of action potentials induced by step‐wise depolarizing current (30 ms; 5 pA increments). (H) The analyses of the rheobase (one‐way ANOVA, interaction *F*_(2,21)_ = 3.845, *p* = 0.0378; Saline vs. CQ: *p* = 0.2694; Saline vs. His: *p* = 0.0301 < 0.05; CQ vs. His: *p* = 0.4857, *n* = 8 neurons per group, mice = 3 per group). (I) The analyses of the resting membrane potential (RMP) (one‐way ANOVA, interaction *F*_(2,21)_ = 5.086, *p* = 0.0158; Saline vs. CQ: *p* = 0.9691; Saline vs. His: *p* = 0.0237 < 0.05; CQ vs. His: *p* = 0.0394 < 0.05, *n* = 8 neurons per group, mice = 3 per group). *p < 0.05, **p < 0.01. CQ, chloroquine; His, histamine.

To investigate the characteristics of the LSN^Tac1^ neurons in response to acute itch stimuli, we employed an electrophysiological approach (Figure 5A). Initially, a significant increase in the amplitude of sEPSCs was observed in the CQ group, whereas no such increase was noted in the His‐ or saline‐injected group (Figure 5B,C). The frequency of sEPSCs showed no significant differences among the CQ, His, and saline groups (Figure 5D). The inhibitory transmission may also contribute to electrophysiological regulation. Future studies will address the potential involvement of inhibitory inputs.

The firing frequency induced by depolarizing step currents increased in mice subjected to acute itch models (Figure 5E,F). During our recordings, we monitored input resistance (Rin) using hyperpolarizing test pulses and did not detect any significant differences among the saline, CQ, and His groups (Figure S6). However, the minimum current required to elicit an AP (Rheobase) decreased dramatically in the His group (Figure 5G,H). The parameters of passive membrane properties, specifically resting membrane potential (RMP), showed significant differences among the groups (His, CQ, and saline) (Figure 5I). This discrepancy may reflect changes in ion channel kinetics or adaptation‐related conductance (e.g., Na^+^ channel availability, A‐type or M‐type K^+^ currents) [22]. The A‐type potassium current is a fast‐inactivating current that delays the onset of action potentials and controls initial firing frequency. In contrast, the M‐type potassium current is a slow, non‐inactivating current that persistently suppresses neuronal excitability to limit sustained firing. However, implementing more precise classification methods would enable finer analysis of neuronal subtype‐specific functional roles, which could significantly enhance mechanistic insights into spinal dorsal horn processing.

Collectively, our data demonstrate that the LSN^Tac1^ neurons process histaminergic and non‐histaminergic itch through fundamentally different mechanisms, with CQ enhancing postsynaptic neuronal activation while His increases intrinsic excitability.

### 3.5 The LSN^Phox2a^ Neurons Primarily Participate in CQ‐Induced Itch Scratching Behavior

**FIGURE 6:**
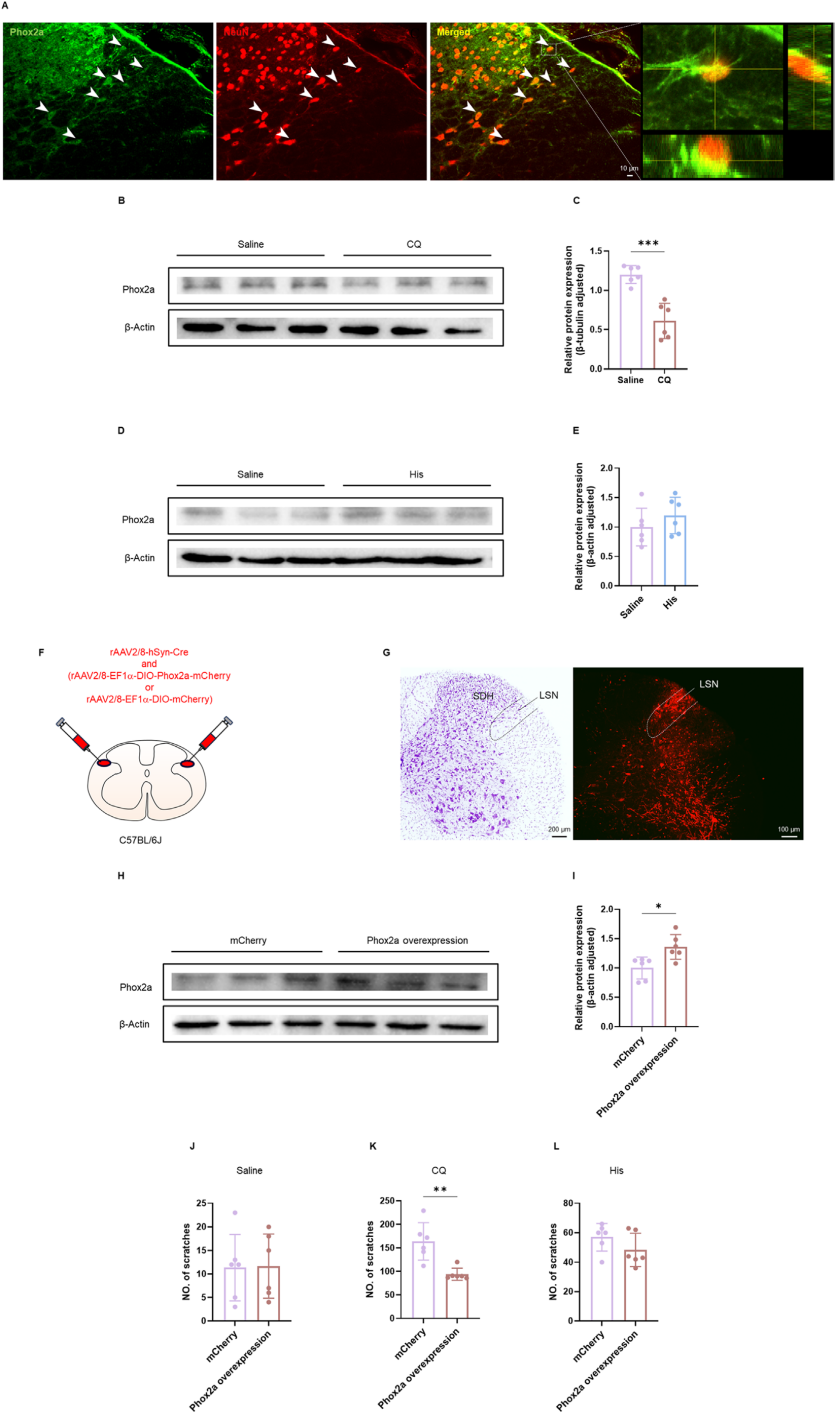
The role of Phox2a in the LSN neurons in CQ‐induced itch. (A) The Phox2a‐immunoreactive neurons (green), NeuN‐immunoreactive neurons (red) and Phox2a/NeuN double‐labeled neurons (merged) were observed in the LSN. The corresponding images of the rectangles were enlarged in (right). (B) Western blotting bands of the Phox2a expressions in saline and CQ groups of mice. (C) Quantitative analysis of the Phox2a, compared to the control (unpaired two‐tailed *t*‐test, *t*_(10)_ = 5.773, *p* = 0.0002 < 0.001, *n* = 6 per group). (D) Western blotting bands of the LSN^Phox2a^ molecules in saline and histamine groups of mice. (E) Quantitative analysis of the LSN^Phox2a^, compared to the control (unpaired two‐tailed *t*‐test, *t*_(10)_ = 1.085, *p* = 0.3033, *n* = 6 per group). (F) Schematic diagram showing injection sites of the rAAV2/8‐hSyn‐Cre and rAAV2/8‐EF1α‐DIO‐Phox2a‐mCherry or rAAV2/8‐EF1α‐DIO‐mCherry into the bilateral side LSN. (G) The injection site of the virus in the LSN (right) with a Nissl staining of the adjacent section (left). (H) Western blotting analyses after overexpression of Phox2a in the LSN. (I) Quantitative analyses results comparing the overexpression of Phox2a levels in the LSN with the control group (unpaired two‐tailed *t*‐test, *t*_(10)_ = 3.141, *p* = 0.0105 < 0.05, *n* = 6 per group). (J–L) Viral overexpression of Phox2a in the LSN neurons had no significant effect on saline‐ or His‐induced scratching behaviors in mice (unpaired two‐tailed *t*‐test, Saline: *t*_(10)_ = 0.08310, *p* = 0.9354; His: *t*_(10)_ = 1.444, *p* = 0.1795; *n* = 6 per group), whereas attenuated CQ‐induced scratching behaviors (unpaired two‐tailed *t*‐test, *t*_(10)_ = 4.104, *p* = 0.0021 < 0.01, *n* = 6 per group). *p < 0.05, **p < 0.01. LSN, lateral spinal nucleus; SDH, spinal dorsal horn.

Phox2a is predominantly expressed in the LSN; therefore, we hypothesized that the LSN^Phox2a^ neurons are involved in itch neurotransmission [23]. We first observed Phox2a expression in the LSN of C57BL/6J mice (Figure 6A). Then, the results demonstrated that the protein levels of Phox2a were reduced in CQ‐treated mice compared to saline controls (Figure 6B,C), with no significant changes observed in His‐induced animals (Figure 6D,E). This CQ‐specific reduction of Phox2a in the LSN neurons suggests a unique mechanism underlying His‐independent itch. Collectively, these findings establish Phox2a as a critical regulator of CQ‐induced itch neurotransmission within the LSN.

Given the downregulation of Phox2a in CQ‐induced itch, we next investigated the effects of Phox2a overexpression in the LSN neurons. We administered rAAV2/8‐EF1α‐DIO‐Phox2a‐mCherry and rAAV2/8‐hSyn‐Cre to overexpress Phox2a in the bilateral LSN of C57BL/6J mice (Figure 6F,G). The rAAV2/8‐EF1α‐DIO‐mCherry and rAAV2/8‐hSyn‐Cre were injected as the control. Western blotting was employed to verify the effectiveness of the virus (Figure 6H,I). Behavioral analyses revealed a marked reduction in CQ‐induced scratching behaviors in the Phox2a overexpression group compared with the mCherry control group, whereas the saline and His group did not exhibit significant changes (Figure 6J–L). The locomotor abilities in the OFT and the anxiety‐like behaviors evaluated using the EPM test remained unaffected by the modulation of the LSN^Phox2a^ neurons (Figure S7A–F). Together, the behavioral experiments suggest that overexpression of the LSN^Phox2a^ neurons alleviates the CQ‐induced itch.

These results demonstrate that the LSN^Phox2a^ neurons selectively mediate CQ‐induced itch through a histamine‐independent pathway.

### 3.6 Phox2a Overexpression Ameliorates CQ‐Induced Scratching Behaviors in the LSN^Tac1^ Neurons

**FIGURE 7:**
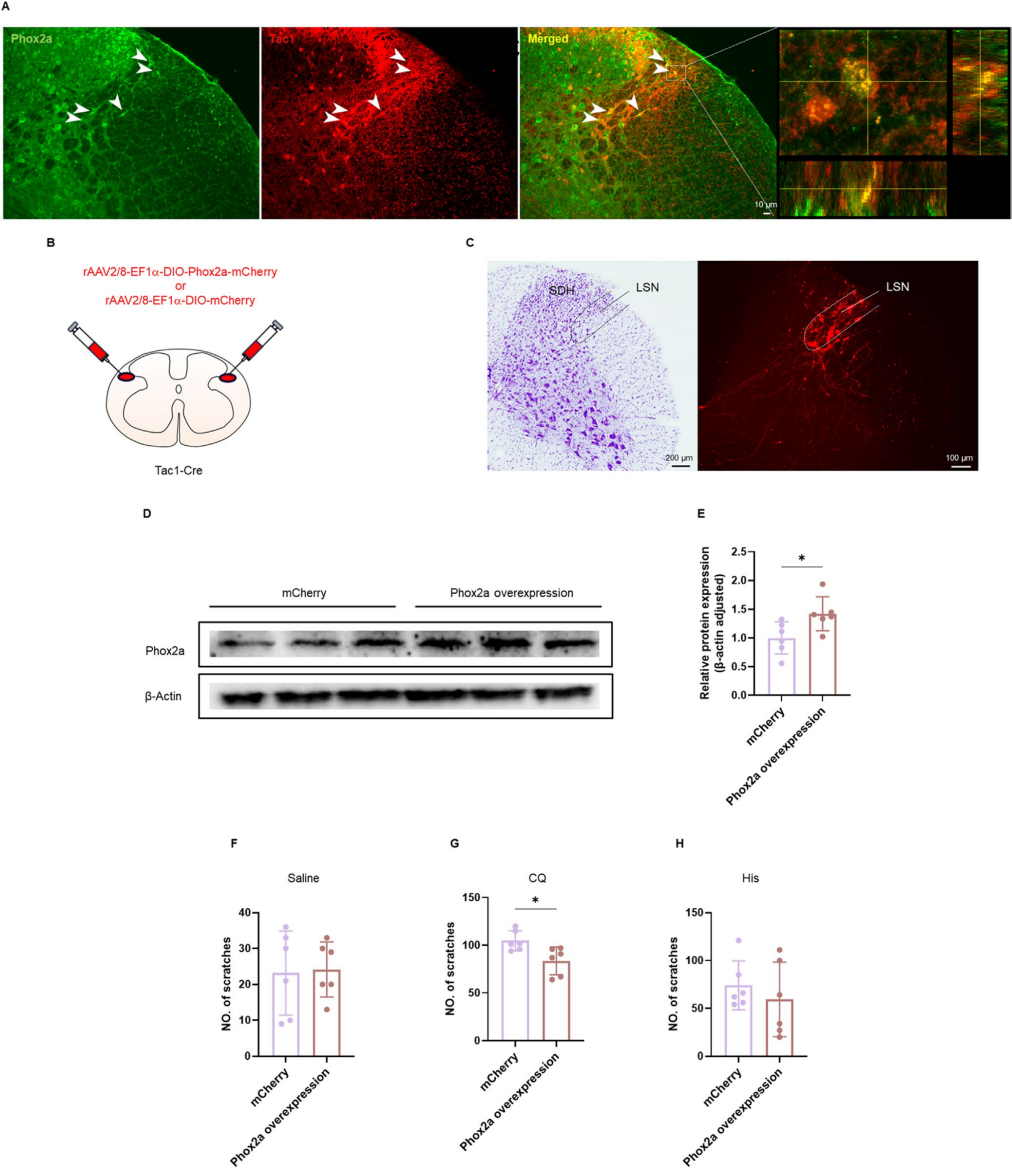
The roles of Phox2a in the LSN^Tac1^ neurons. (A) Phox2a‐immunoreactive neurons (green), tdTomato‐labeled Tac1‐positive neurons (red) and the Phox2a/Tac1 double‐labeled neurons (merged) were observed in the LSN. The corresponding images of the rectangles were enlarged in (right). (B) Schematic diagram showing injection sites of the rAAV2/8‐EF1α‐DIO‐Phox2a‐mCherry or rAAV2/8‐EF1α‐DIO‐mCherry into the bilateral side LSN. (C) The injection site of the virus in the LSN (right) with a Nissl staining of the adjacent section (left). (D) Western blotting analyses after overexpression of Phox2a in the LSN^Tac1^ neurons. (E) Quantitative analysis results comparing the overexpression of Phox2a levels in the LSN^Tac1^ neurons with the control group (unpaired two‐tailed *t*‐test, *t*_(10)_ = 2.533, *p* = 0.0297 < 0.05, *n* = 6 per group). (F–H) Viral overexpression of Phox2a in the LSN^Tac1^ neurons had no significant effect on saline‐ or His‐induced scratching behaviors in mice (unpaired two‐tailed *t*‐test, Saline: *t*_(10)_ = 0.1748, *p* = 0.8647; His: *t*_(10)_ = 0.7715, *p* = 0.4582; *n* = 6 per group), whereas attenuated CQ‐induced scratching behaviors (unpaired two‐tailed *t*‐test, *t*_(10)_ = 2.866, *p* = 0.0168 < 0.05, *n* = 6 per group). *p < 0.05. LSN, lateral spinal nucleus; SDH, spinal dorsal horn.

Our previous findings demonstrated the effects of modulations of the LSN^Phox2a^ neurons in CQ‐induced itch, leading us to investigate whether this regulatory effect specifically occurred in *Tac1*‐positive neurons. Immunofluorescence staining in Tac1‐Ai9 mice revealed the Phox2a expression in *Tac1*‐positive neurons within the LSN (Figure 7A). Consequently, we injected a Cre‐dependent anterograde virus (rAAV‐EF1α‐DIO‐Phox2a‐mCherry) to overexpress Phox2a in the bilateral LSN of Tac1‐Cre mice, with rAAV‐EF1α‐DIO‐mCherry serving as the control (Figure 7B,C). Western blotting was employed to verify the effectiveness of the virus (Figure 7D,E). The results revealed that the overexpression of Phox2a in the LSN^Tac1^ neurons had no impact on saline‐ or His‐induced scratching behaviors (Figure 7F,H). The CQ model showed significantly decreased scratching behaviors following Phox2a overexpression (Figure 7G).

The locomotor abilities in the OFT and the anxiety‐like behaviors remained unaffected by overexpression of Phox2a in the LSN^Tac1^ neurons (Figure S7G–L). Together, these results suggest that Phox2a overexpression in the LSN^Tac1^ neurons reduces CQ‐induced scratching behaviors but does not affect His‐induced itch.

Therefore, our findings indicate that the overexpression of Phox2 may play a crucial role in braking the transmission of itch information induced by CQ in the LSN^Tac1^ neurons.

### 3.7 Phox2a in the LSN^Tac1^ Neurons Differentially Modulates Electrophysiological Responses to CQ‐ and his‐Induced Itch

To investigate the electrophysiological consequences of Phox2a overexpression in the LSN^Tac1^ neurons, we injected Tac1‐Cre mice with either rAAV2/8‐EF1α‐DIO‐Phox2a‐mCherry (experimental group) or rAAV2/8‐EF1α‐DIO‐mCherry (control group) into the LSN (Figure 7B, Figure S8A). Whole‐cell patch‐clamp recordings revealed a significant reduction in both the amplitude and frequency of sEPSCs in Phox2a‐overexpressing neurons compared to controls (Figure S8B–D). Notably, intrinsic electrophysiological properties remained unaltered. Specifically, no significant differences were detected between groups in AP firing rates evoked by depolarizing currents, rheobase or RMP (Figure S8E–I). These results indicate that Phox2a exerts its effects by reducing presynaptic neurotransmitter release and decreasing postsynaptic neuronal excitability in the LSN^Tac1^ neurons.

**FIGURE 8:**
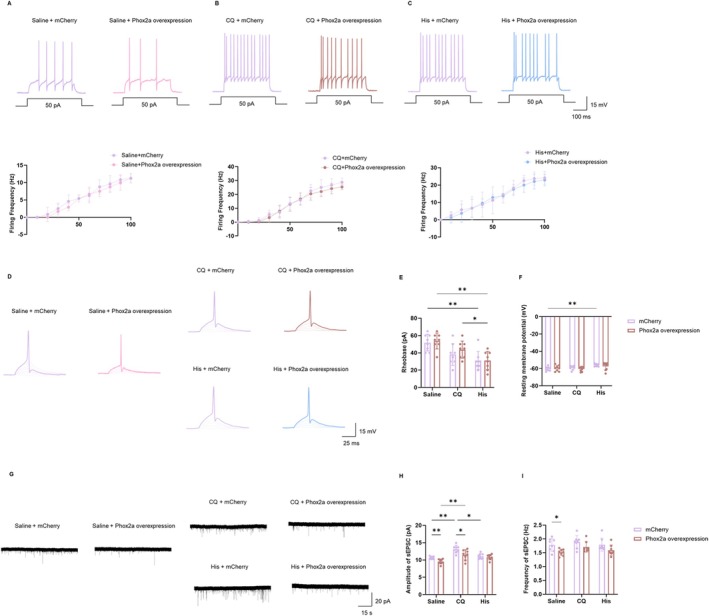
Phox2a overexpression in the LSN^Tac1^ neurons differentially modulates electrophysiological responses to CQ‐ and His‐induced itch. (A–C) Representative traces (upper panels) and line charts (lower panels) show the changes in firing frequency of the LSN^Tac1^ neurons in different groups (saline: Two‐way ANOVA, interaction *F*_(10,154)_ = 0.6971, *p* = 0.7261; CQ: Two‐way ANOVA, interaction *F*_(10,154)_ = 1.700, *p* = 0.0852; His: Two‐way ANOVA, interaction *F*_(10,154)_ = 0.2135, *p* = 0.9948; *n* = 6 neurons per group, mice = 3 per group). (D) Sample traces of action potentials induced by step‐wise depolarizing current (30 ms; 5 pA increments). (E) The analysis of the rheobase (two‐way ANOVA, interaction *F*_(2,28)_ = 0.3672, *p* = 0.6960; mCherry: Saline vs. His: *p* = 0.0035; Phox2a overexpression: Saline vs. His: *p* = 0.0020 < 0.01; CQ vs. His: *p* = 0.0127, *n* = 8 neurons per group, mice = 3 per group). (F) The analysis of the resting membrane potential (RMP) (two‐way ANOVA, interaction *F*_(2,28)_ = 0.7412, *p* = 0.4857; mCherry: Saline vs. His: *p* = 0.0025 < 0.01, *n* = 8 neurons per group, mice = 3 per group). (G) Sample traces of sEPSC recorded at −70 mV from mCherry‐control and Phox2a‐overexpressing neurons in the Saline, CQ, and His groups. (H) Summary data of the amplitude of sEPSC (two‐way ANOVA, interaction *F*_(2,28)_ = 2.112, *p* = 0.1399; Saline: mCherry vs. Phox2a overexpression: *p* = 0.0028 < 0.01; CQ: mCherry vs. Phox2a overexpression: *p* = 0.0231 < 0.05; mCherry: Saline vs. CQ: *p* = 0.0016 < 0.01; CQ vs. His: *p* = 0.0152 < 0.05; Phox2a overexpression: Saline vs. CQ: *p* = 0.0069 < 0.01; *n* = 8 neurons per group; mice = 3 per group). (I) Summary data of the frequency of sEPSC (two‐way ANOVA, interaction *F*_(2,28)_ = 0.1759, *p* = 0.8396; Saline: mCherry vs. Phox2a overexpression: *p* = 0.0142 < 0.01; *n* = 8 neurons per group; mice = 3 per group). *p < 0.05, **p < 0.01. CQ, chloroquine; His, histamine.

The effects of Phox2a overexpression selectively attenuated CQ‐ but not His‐induced itch behaviors; therefore, we compared the electrophysiological properties of the LSN^Tac1^ neurons under three conditions: His‐dependent itch, His‐independent (CQ) itch, and saline control. Whole‐cell recordings showed no significant difference in intrinsic excitability parameters, such as AP firing rates, rheobase, and RMP, between the experimental and control groups (Figure 8A–D). However, His stimulation resulted in a lower rheobase and a slight depolarization of RMP in mCherry‐expressing controls relative to saline controls (Figure 8E,F). RMP did not differ significantly between Phox2a‐overexpressing and mCherry‐expressing LSN^Tac1^ neurons in the His group.

Importantly, during CQ itch, Phox2a‐overexpressing LSN^Tac1^ neurons exhibited a selective reduction in sEPSC amplitude, but not frequency, compared to mCherry‐expressing controls (Figure 8G–I).

These findings suggest that, unlike His‐dependent itch, Phox2a likely mediates the attenuation of CQ‐induced scratching behaviors through the suppression of postsynaptic neuronal excitability in the LSN^Tac1^ neurons. Conversely, decreased Phox2a expression in the context of CQ‐induced itch may enhance postsynaptic neuronal excitability and thereby contribute to the exacerbated itch behaviors.

## 4 Discussion

In the present study, we demonstrated that the LSN^Tac1^ neurons are activated by acute itch stimuli, particularly in responses to the CQ‐induced itch. Chemogenetic activation of the LSN^Tac1^ neurons significantly increased scratching behaviors in mice, whereas inhibition reduced scratches. Electrophysiological recordings revealed that CQ and His stimuli differentially elevate the excitability of the LSN^Tac1^ neurons. Notably, Phox2a expressed in the LSN^Tac1^ neurons was distinctly reduced in CQ‐ but not His‐induced itch. Critically, overexpression of Phox2a in the LSN^Tac1^ neurons substantially reduced CQ‐induced scratching behaviors, demonstrating that Phox2a plays an essential role in modulating CQ‐induced itch neurotransmission in the LSN^Tac1^ neurons.

The LSN serves to integrate peripheral sensory inputs within the ALS [24, 25, 26]. While prior studies established its role in processing noxious thermal/mechanical stimuli and relaying nociceptive signals from deep tissues to higher brain centers [27, 28, 29, 30, 31, 32], the mechanisms underlying LSN‐mediated itch processing remain poorly characterized. To address this question, we selectively modulated the LSN neuronal activity via chemogenetic manipulation. Notably, the LSN neurons' activation potentiated pruritogen‐induced scratching behaviors, whereas its inhibition reduced these responses. These results identify the roles of the LSN neurons in itch neurotransmission.

The LSN neurons project to somatosensory‐processing regions including the brainstem, thalamus, periaqueductal gray (PAG), and lateral parabrachial nucleus (LPB) [33]. While morphological studies also suggest the LSN connectivity with emotion‐related nuclei (e.g., hypothalamus, thalamus, raphe nucleus) [34, 35, 36, 37]. Our behavioral tests revealed no significant differences in anxiety‐like behaviors: neither OFT central areas duration nor EPM open arm time/total crossings number of times differed between LSN‐chemomanipulated and control mice. Combined with these results, we speculate that the distinct subpopulations of the LSN neurons, which project to different brain regions, may underlie this dissociation, with sensory and emotional‐related circuits potentially segregated across these subpopulations.

Tac1‐expressing neurons are recognized to mediate itch neurotransmission in rodents [38, 39]. While prior research has focused on Tac1‐positive neurons within the ventrolateral PAG (vlPAG) and their roles in facilitating itch processing [40]. in this study, we investigate the distinct roles of Tac1‐expressing neurons in the LSN, which is a key relay hub in the ascending sensory pathway. Our findings revealed that these LSN^Tac1^ neurons are excitatory neurons. Notably, prior research has indicated that LSN neurons co‐express gastrin‐releasing peptide (GRP), a neuropeptide confirmed to be specifically involved in itch neurotransmission in the spinal dorsal horn (SDH). Moreover, these LSN^GRP^ neurons were found to co‐express *Tac1* mRNA. This co‐expression profile suggests that LSN^Tac1^ neurons may represent a specialized subpopulation dedicated to itch transmission within the SDH [38, 39, 41]. Our behavioral data further suggested that the activation of the LSN^Tac1^ neurons amplifies pruritogen‐induced scratching behaviors, while their inhibition reduces the itch behaviors. Consequently, all the aforementioned results collectively confirm the activating roles of the LSN^Tac1^ neurons in the neurotransmission of itch.

Acute itch is mechanistically classified into His‐dependent and His‐independent subtypes [42, 43]. Although effective treatments are available for His‐dependent itch, therapies for His‐independent itch remain inadequate [44]. By uncovering the distinct mechanisms underlying these subtypes, we can improve our understanding of itch signaling and develop more precise diagnostic and therapeutic strategies for itch‐related conditions.

Our study revealed that there exist differences between CQ‐ and His‐induced itch. First, compared with His, the number of c‐FOS was increased significantly within the LSN neurons in the CQ group. Next, we employed electrophysiological recordings to characterize the activity patterns of the LSN^Tac1^ neurons in the CQ or His‐induced itch. The analyses revealed the distinct excitatory mechanisms in the LSN^Tac1^ neurons under CQ‐ and His‐induced itch. While both stimuli increased firing frequency under equivalent current intensities, His elevated RMP and reduced rheobase current, indicating increased intrinsic excitability. In contrast, CQ significantly increased sEPSC amplitudes, which may reflect enhanced postsynaptic responsiveness, potentially arising from alterations in postsynaptic receptor properties or density. In our recordings, although both parameters trended toward enhanced excitability, their magnitudes did not fully overlap across groups, suggesting that additional ionic mechanisms (e.g., changes in sodium/potassium channel properties or other conductance) may differentially contribute to these readouts [22].

Recent studies indicate that Phox2a specifically expressed in the LSN neurons, is essential for spinal‐thalamic circuit development, as its ablation in cervical spinal cord neurons disrupts connectivity and impairs nociceptive transmission [9]. Our data confirm Phox2a expression in the LSN neurons and demonstrate a significant reduction in Phox2a levels following CQ‐treated mice, contrasting with unchanged expression in His‐treated mice. Notably, overexpression of LSN^Phox2a^ selectively reduced CQ‐induced scratching behaviors without affecting His‐induced responses, which is consistent with our Western blotting data. Collectively, these results establish the LSN^Phox2a^ neurons as key mediators of CQ‐induced itch, functionally distinct from His‐dependent pruriceptive mechanisms.

Building on these observations, our morphological data further reveal that the LSN^Tac1^ neurons express Phox2a, implicating this transcription factor in the functional contribution of the LSN^Tac1^ neurons to itch sensation. To test this hypothesis, we selectively overexpressed Phox2a in both LSN and LSN^Tac1^ neurons, observing a significant suppression of CQ‐induced scratching behaviors without altering His‐dependent responses. Our demonstration that Phox2a overexpression alleviates CQ‐induced itch indicates its therapeutic potential but prompts the question of how this target might be modulated pharmacologically. Fortunately, the upstream signaling pathways regulating Phox2a transcription are well characterized, offering drug development opportunities. Pioneering work has established that combined cyclic adenosine monophosphate (cAMP) and bone morphogenetic protein (BMP) signaling synergistically induces Phox2a expression [45], a mechanism subsequently shown to be mediated by the direct binding of cAMP response element‐binding protein (CREB) to a conserved cis‐acting element in the *Phox2a* promoter [46]. This reveals a promising pharmacological strategy: small molecule agonists that elevate cAMP levels (e.g., phosphodiesterase inhibitors) or potentiate BMP signaling could be leveraged to enhance endogenous Phox2a expression in LSN^Tac1^ neurons. This approach would present a clinically translatable avenue for treating His‐independent itch. Future studies could test whether established US Food and Drug Administration (FDA)–approved phosphodiesterase inhibitors (e.g., apremilast) or BMP pathway agonists can mimic the anti‐pruritic effects with Phox2a overexpression, thereby repurposing these agents or their derivatives as novel itch therapeutics.

We found that His increased the intrinsic excitability of LSN^Tac1^ neurons, while CQ enhanced postsynaptic neuronal activation. To further investigate this, we overexpressed Phox2a in the LSN^Tac1^ neurons and examined its effects on sEPSCs and intrinsic excitability. Results showed that Phox2a overexpression specifically reduced the amplitude and frequency of sEPSCs in the LSN^Tac1^ neurons, whereas intrinsic excitability parameters, including AP firing rates, rheobase, and RMP remained unaffected.

Next, we examined the effects of Phox2a overexpression on sEPSCs in itch models. Results demonstrated that in CQ‐treated mice, Phox2a overexpression significantly reduced sEPSC amplitude compared to mCherry‐expressing controls, indicating suppression of postsynaptic neuronal activation specifically targeting His‐independent pathways. In contrast, His‐induced synaptic transmission remained unchanged, with no significant alterations in sEPSC amplitude or frequency observed between Phox2a‐overexpressing and control groups. These differential synaptic effects likely reflect the specific roles of Phox2a in CQ‐induced itch.

Intriguingly, under saline conditions, the finding that Phox2a overexpression alone reduced both sEPSC amplitude and frequency suggests a diminished excitatory synaptic drive, which may involve both presynaptic and postsynaptic mechanisms. However, in the CQ‐induced itch model, Phox2a overexpression selectively decreased sEPSC amplitude without altering its frequency. The results under CQ stimulation suggest distinct regulatory mechanisms for synaptic modulation, which warrant further investigation.

Notably, compared with saline groups, mice exhibited an elevation of the RMP in response to His. However, no significant difference was observed between the control virus and the Phox2a‐overexpressing LSN^Tac1^ neurons. Phox2a selectively mediates CQ‐induced itch transmission in LSN^Tac1^ neurons, with no observed effect on His‐mediated effects.

## 5 Conclusions

In summary, our study illustrates that Phox2a in the LSN^Tac1^ neurons primarily mediates CQ‐induced itch neurotransmission. We demonstrate that CQ downregulates Phox2a in these neurons, and that Phox2a overexpression effectively ameliorates itch‐induced scratching by reducing postsynaptic neuronal excitability. These findings reveal novel insights into the mechanism underlying Phox2a‐mediated regulation of His‐independent acute itch. This discovery not only provides a potential therapeutic target for spinal‐level antipruritic drug development, but also proposes a mechanism‐based strategy to differentiate between distinct subtypes of pruritic disorders.

## Author Contributions

The first draft of the manuscript was performed by Y.‐L.C.; electrophysiological experiments were performed by Z.‐A.L.; virus injections and data collection were performed by Y.‐L.C.; Western blotting was performed by Y.‐L.C. and Q.‐Z.W.; statistical analysis was performed by E.M.; Y.‐H.L., X.‐R.W., Z.‐Z.K., and Y.‐Q.L. mainly contributed to the study conception and design. Z.‐P.C., Z.‐Z.K., and Y.‐Q.L. also revised the manuscript. All authors read and approved the final manuscript.

## Ethics Statement

The study was approved by the Ethics Committee of the Laboratory Animal Center at the Fourth Military Medical University (ethics approval number: IACUC‐20210356) to minimize animal suffering. The care and use of experimental animals strictly followed institutional guidelines and government regulations.

## Conflicts of Interest

The authors declare no conflicts of interest.

## Supporting information


**Figures S1–S8.** cns70639‐sup‐0001‐FigureS1‐S8.zip.

## Data Availability

The data provided in this study are available on request from the corresponding author. These data are not publicly available due to the privacy of other scientific studies.
