# Supplementary figures and images for: Phox2a in Lateral Spinal Nucleus Tac1‐Positive Neurons Mediates Histamine‐Independent Acute Itch
Source: CNS Neurosci Ther. 2025 Nov 7;31(11):e70639. doi: 10.1111/cns.70639 (PMC12594609; doi:10.1111/cns.70639)

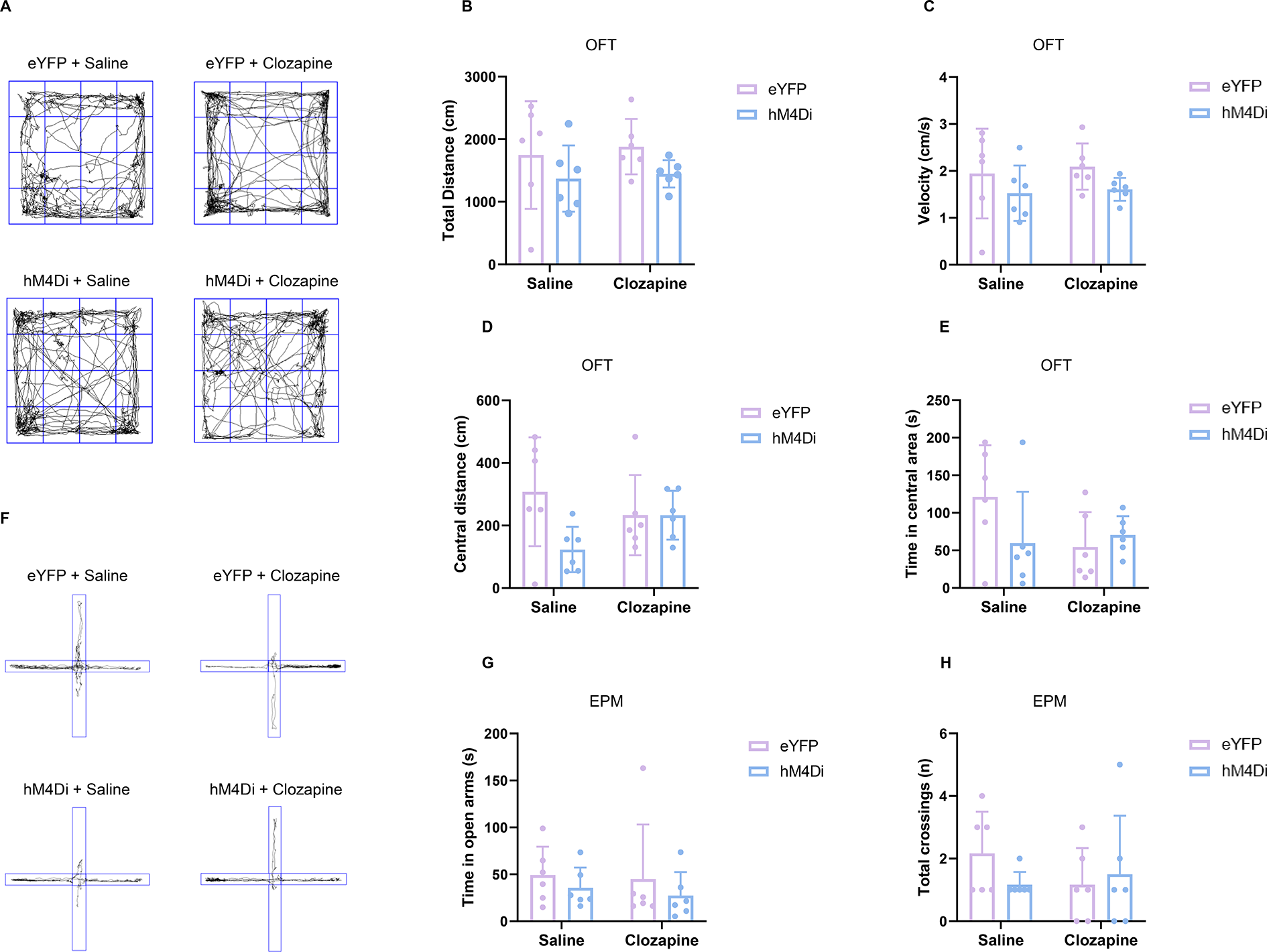

Supplement: Supplementary file 1 — Figures S1–S8. cns70639‐sup‐0001‐FigureS1‐S8.zip. [file CNS-31-e70639-s001.zip › cns70639-sup-0001-FigureS1-S8/cns70639-sup-0006-FigureS5-S5.tif]
