# Supplementary material for: Phox2a in Lateral Spinal Nucleus Tac1‐Positive Neurons Mediates Histamine‐Independent Acute Itch
Source: CNS Neurosci Ther. 2025 Nov 7;31(11):e70639. doi: 10.1111/cns.70639 (PMC12594609; doi:10.1111/cns.70639)
Supplement: Supplementary file 1 — Figures S1–S8. cns70639‐sup‐0001‐FigureS1‐S8.zip. [file CNS-31-e70639-s001.zip › cns70639-sup-0001-FigureS1-S8/cns70639-sup-0002-FigureS1-S8.doc]

**Supplementary Materials**

**Supplementary Figures**

**Fig. S1** Acute itch and acute pain stimulation induce scratching behavior that is correlated with neuronal activation in the LSN. **A** Quantification of scratching bouts over a 30-minute period following intradermal injection of saline, Cap, CQ, or His. Data are presented as mean ± S.D.. (one-way ANOVA, F_(3,16)_ = 90.99, *p* < 0.0001; Saline *vs.* CQ: *p* < 0.0001; Saline *vs.* His: *p* = 0.001; *n* = 5 per group). **B** Scatter plot showing a strong positive correlation between the number of scratching bouts and the number of c-FOS-positive neurons in the LSN across all treatment groups. (Pearson correlation, R² = 0.98, *p* < 0.0001). Each data point represents the average value for one treatment group. ****P* < 0.001, *****p* < 0.0001. CQ: chloroquine; His: histamine; Cap: capsaicin.

**Fig. S2** Chemogenetic activation of the LSN neurons has no effect on acute itch-induced anxiety-like behaviors. **A** Illustrative movement traces from the four groups in the OFT. **B**-**E** Effects of chemogenetic activation of the LSN neurons on locomotor abilities and anxiety-like behaviors in the OFT (two-way ANOVA, total distances: interaction F_(1,20)_ = 1.411, *p* = 0.2489; eGFP-Saline *vs.* hM3Dq-Clozapine: *p* = 0.1007; hM3Dq-Saline *vs.* hM3Dq-Clozapine: *p* = 0.3061; eGFP-Clozapine *vs.* hM3Dq-Clozapine: *p* = 0.1242; velocity: interaction F_(1,20)_ = 1.404, *p* = 0.2500; eGFP-Saline *vs.* hM3Dq-Clozapine: *p* = 0.1012; hM3Dq-Saline *vs.* hM3Dq-Clozapine: *p* = 0.3081; eGFP-Clozapine *vs.* hM3Dq-Clozapine: *p* = 0.1247; central distance: interaction F_(1,20)_ = 1.598, *p* = 0.2208; eGFP-Saline *vs.* hM3Dq-Clozapine: *p* = 0.3816; hM3Dq-Saline *vs.* hM3Dq-Clozapine: *p* = 0.2836; eGFP-Clozapine *vs.* hM3Dq-Clozapine: *p* = 0.4107; time in center areas: interaction F_(1,20)_ = 0.4844, *p* = 0.4945; eGFP-Saline *vs.* hM3Dq-Clozapine: *p* = 0.9978; hM3Dq-Saline *vs.* hM3Dq-Clozapine: *p* = 0.9260; eGFP-Clozapine *vs.* hM3Dq-Clozapine: *p* = 0.9976; *n* = 6 per group). **F** Illustrative movement traces from the four groups in the EPM. **G**-**H** Effects of chemogenetic activation of the LSN neurons on anxiety-like behaviors in the EPM (two-way ANOVA, time in open arms: interaction F_(1,20)_ = 0.5219, *p* = 0.4784; eGFP-Saline *vs.* hM3Dq-Clozapine: *p* = 0.8833; hM3Dq-Saline *vs.* hM3Dq-Clozapine: *p* = 0.2943; eGFP-Clozapine *vs.* hM3Dq-Clozapine: *p* = 0.9999; total crossing: interaction F_(1,20)_ = 0.03810, *p* = 0.8472; eGFP-Saline *vs.* hM3Dq-Clozapine: *p* = 0.9982; hM3Dq-Saline *vs.* hM3Dq-Clozapine: *p* = 0.9997; eGFP-Clozapine *vs.* hM3Dq-Clozapine: *p* > 0.9999; *n* = 6 per group). OFT: open field test; EPM: elevated plus maze test.

**Fig. S3** Chemogenetic inhibition of the LSN neurons has no effect on acute itch-induced anxiety-like behaviors. **A** Illustrative movement traces from the four groups in the OFT task. **B**-**E** Effects of chemogenetic inhibition of the LSN neurons on locomotor abilities and anxiety-like behaviors in the OFT (two-way ANOVA, total distances: interaction F_(1,20)_ = 0.2443, *p* = 0.6265; eGFP-Saline *vs.* hM4Di-Clozapine: *p* = 0.9724; hM4Di-Saline *vs.* hM4Di-Clozapine: *p* = 0.9326; eGFP-Clozapine *vs.* hM4Di-Clozapine: *p* = 0.9493; velocity: interaction F_(1,20)_ = 0.2452, *p* = 0.6258; eGFP-Saline *vs.* hM4Di-Clozapine: *p* = 0.9724; hM4Di-Saline *vs.* hM4Di-Clozapine: *p* = 0.9320; eGFP-Clozapine *vs.* hM4Di-Clozapine: *p* = 0.9494; central distance: interaction F_(1,20)_ = 1.695, *p* = 0.2078; eGFP-Saline *vs.* hM4Di-Clozapine: *p* = 0.2887; hM4Di-Saline *vs.* hM4Di-Clozapine: *p* = 0.3140; eGFP-Clozapine *vs.* hM4Di-Clozapine: *p* = 0.2607; time in center areas: interaction F_(1,20)_ = 1.706, *p* = 0.2064; eGFP-Saline *vs.* hM4Di-Clozapine: *p* = 0.2406; hM4Di-Saline *vs.* hM4Di-Clozapine: *p* = 0.1667; eGFP-Clozapine *vs.* hM4Di-Clozapine: *p* = 0.3865; *n* = 6 per group). **F** Illustrative movement traces from the four groups in the EPM task. **G**-**H** Effects of chemogenetic inhibition of the LSN neurons on anxiety-like behaviors in the EPM (two-way ANOVA, time in open arms: interaction F_(1,20)_ = 0.1968, *p* = 0.6621; eGFP-Saline *vs.* hM4Di-Clozapine: *p* = 0.9012; hM4Di-Saline *vs.* hM4Di-Clozapine: *p* = 0.3843; eGFP-Clozapine *vs.* hM4Di-Clozapine: *p* =  0.9885; total crossings: interaction F_(1,20)_ = 1.613, *p* = 0.2187; eGFP-Saline *vs.* hM4Di-Clozapine: *p* = 0.7717; hM4Di-Saline *vs.* hM4Di-Clozapine: *p* = 0.3886; eGFP-Clozapine *vs.* hM4Di-Clozapine: *p* = 0.6728; *n* = 6 per group). OFT, open field test; EPM, elevated plus maze test.

**Fig. S4** Chemogenetic activation of the LSN^Tac1^ neurons has no effect on acute itch-induced anxiety-like behaviors. **A** Illustrative movement traces from the four groups in the OFT. **B**-**E** Effects of chemogenetic activation of the LSN^Tac1^ neurons on locomotor abilities and anxiety-like behaviors in the OFT (two-way ANOVA, total distances: interaction F_(1,20)_ = 4.036, *p* = 0.0582; eYFP-Saline *vs.* hM3Dq-Clozapine: *p* = 0.5938; hM3Dq-Saline *vs.* hM3Dq-Clozapine: *p* = 0.1073; eYFP-Clozapine *vs.* hM3Dq-Clozapine: *p* = 0.3511; velocity: interaction F_(1,20)_ = 4.038, *p* = 0.0582; eYFP-Saline *vs.* hM3Dq-Clozapine: *p* = 0.5933; hM3Dq-Saline *vs.* hM3Dq-Clozapine: *p* = 0.1072; eYFP-Clozapine *vs.* hM3Dq-Clozapine: *p* = 0.3506; central distance: interaction F_(1,20)_ = 0.07951, *p* = 0.7809; eYFP-Saline *vs.* hM3Dq-Clozapine: *p* = 0.2094; hM3Dq-Saline *vs.* hM3Dq-Clozapine: *p* = 0.5255; eYFP-Clozapine *vs.* hM3Dq-Clozapine: *p* = 0.7217; time in center areas: interaction F_(1,20)_ = 4.103, *p* = 0.0564; eYFP-Saline *vs.* hM3Dq-Clozapine: *p* = 0.4405; hM3Dq-Saline *vs.* hM3Dq-Clozapine: *p* = 0.8010; eYFP-Clozapine *vs.* hM3Dq-Clozapine: *p* = 0.9728; *n* = 6 per group). **F** Illustrative movement traces from the four groups in the EPM. **G**-**H** Effects of chemogenetic activation of the LSN^Tac1^ neurons on anxiety-like behaviors in the EPM tests (two-way ANOVA, time in open arms: interaction F_(1,20)_ = 2.145, *p* = 0.1586; eYFP-Saline *vs.* hM3Dq-Clozapine: *p* = 0.2558; hM3Dq-Saline *vs.* hM3Dq-Clozapine: *p* = 0.1408; eYFP- Clozapine *vs.* hM3Dq-Clozapine: *p* = 0.3410; total crossing: interaction F_(1,20)_ = 0.6061, *p* = 0.4454; eYFP-Saline *vs.* hM3Dq-Clozapine: *p* = 0.0550; hM3Dq-Saline *vs.* hM3Dq-Clozapine: *p* = 0.0550; eYFP-Clozapine *vs.* hM3Dq-Clozapine: *p* = 0.6930; *n* = 6 per group). ****P* < 0.001, *****p* < 0.0001. OFT, open field test; EPM, elevated plus maze test.

**Fig. S5** Chemogenetic inhibition of the LSN^Tac1^ neurons has no effect on acute itch-induced anxiety-like behaviors. **A** Illustrative movement traces from the four groups in the OFT. **B**-**E** Effects of chemogenetic inhibition of the LSN^Tac1^ neurons on locomotor abilities and anxiety-like behaviors in the OFT (two-way ANOVA, total distances: interaction F_(1,20)_ = 0.0160, *p* = 0.9006; eYFP-Saline *vs.* hM4Di-Clozapine: *p* = 0.7885; hM4Di-Saline *vs.* hM4Di-Clozapine: *p* = 0.9955; eYFP-Clozapine *vs.* hM4Di-Clozapine: *p* = 0.5490; velocity: interaction F_(1,20)_ = 0.01609, *p* = 0.9003; eYFP-Saline *vs.* hM4Di-Clozapine: *p* = 0.7882; hM4Di-Saline *vs.* hM4Di-Clozapine: *p* = 0.9956; eYFP-Clozapine *vs.* hM4Di-Clozapine: *p* = 0.5485; central distance: interaction F_(1,20)_ = 3.491, *p* = 0.0764; eYFP-Saline *vs.* hM4Di-Clozapine: *p* = 0.7091; hM4Di-Saline *vs.* hM4Di-Clozapine: *p* = 0.4141; eYFP-Clozapine *vs.* hM4Di-Clozapine: *p* > 0.9999; time in center areas: interaction F_(1,20)_ = 2.970, *p* = 0.1002; eYFP-Saline *vs.* hM4Di-Clozapine: *p* = 0.4021; hM4Di-Saline *vs.* hM4Di-Clozapine: *p* = 0.9865; eYFP-Clozapine *vs.* hM4Di-Clozapine: *p* = 0.9575; *n* = 6 per group). **F** Illustrative movement traces from the four groups in the EPM. **G**-**H** Effects of chemogenetic inhibition of the LSN^Tac1^ neurons on anxiety-like behaviors in the EPM tests (two-way ANOVA, time in open arms: interaction F_(1,20)_ = 0.01812, *p* = 0.8943; eYFP-Saline *vs.* hM4Di-Clozapine: *p* = 0.7355; hM4Di-Saline *vs.* hM4Di-Clozapine: *p* = 0.9799; eYFP-Clozapine *vs.* hM4Di-Clozapine: *p* = 0.8387; total crossings: interaction F_(1,20)_ = 1.569, *p* = 0.2249; eYFP-Saline *vs.* hM4Di-Clozapine: *p* = 0.8123; hM4Di-Saline *vs.* hM4Di-Clozapine: *p* = 0.9703; eYFP-Clozapine *vs.* hM4Di-Clozapine: *p* = 0.9703; *n* = 6 per group). OFT, open field test; EPM, elevated plus maze test.

**Fig. S6** Membrane input resistance (Rin) of neurons from Saline, CQ, and His treatment groups (one-way ANOVA, interaction F_(2,21)_ = 3.426, *p* = 0.0515, *n* = 8 neurons per group, mice = 3 per group). His: histamine; CQ: chloroquine.

**Fig. S7** Overexpression of Phox2a in the LSN and the LSN^Tac1^ neurons does not affect acute itch-induced anxiety-like behaviors. **A**-**D** Effects of injection overexpression of Phox2a virus in the LSN neurons on locomotor abilities and anxiety-like behaviors in the OFT (unpaired two-tailed t-test, total distances: t_(10)_ = 0.7995, *p* = 0.4426; velocity: t_(10)_ = 0.8002, *p* = 0.4422; central distance: t_(10)_ = 0.4256, *p* = 0.6794; time in center areas: t_(10)_ = 0.04718, *p* = 0.9633; *n* = 6 per group). **E** and **F** Effects of injection overexpression of Phox2a virus in the LSN on anxiety-like behaviors in the EPM test (unpaired two-tailed t-test, time in open arms: t_(10)_ = 1.540, *p* = 0.1546; total crossings: t_(10)_ = 0.2118, *p* = 0.8365; *n* = 6 per group). **G**-**J** Effects of injection overexpression Phox2a virus of the LSN^Tac1^ neurons on locomotor abilities and anxiety-like behaviors in the OFT (unpaired two-tailed t-test, total distances: t_(10)_ = 1.597, *p* = 0.1413; velocity: t_(10)_ = 1.597, *p* = 0.1413; central distance: t_(10)_ = 1.065, *p* = 0.3121; time in center areas: t_(10)_ = 0.7071, *p* = 0.4956; *n* = 6 per group). **K** and **L** Effects of overexpression Phox2a in the LSN^Tac1^ neurons on anxiety-like behaviors in the EPM tests (unpaired two-tailed t-test, time in open arms: t_(10)_ = 0.7565, *p* = 0.4668; total crossings: t_(10)_ = 0.2351, *p* = 0.8189; *n* = 6 per group). OFT: open field test; EPM: elevated plus maze test.

**Fig. S****8** Phox2a overexpression alters intrinsic electrophysiological properties of the LSN^Tac1^ neurons. **A** Representative confocal image showing co-localization of mCherry (red, viral-labeled Tac1 neurons) and biocytin (green, recorded neuron). **B** Sample traces of sEPSC. **C** and **D** Summary data of the amplitude and the frequency of sEPSC (unpaired t test, amplitude: t = 3.205, *p* = 0.0064 < 0.01; frequency: t = 3.758, *p* = 0.0021 < 0.01; *n* = 8 neurons per group; mice = 3 per group). **E** Sample traces of action potential induced by depolarizing current (400 ms; 5 pA increments). **F** Summary data of the firing frequency induced by step-wise depolarizing currents (two-way ANOVA, interaction F_(20,294)_ = 0.4467, *p* = 0.9821, *n* = 8 neurons per group, mice = 3 per group). **G** Sample traces of action potentials induced by step-wise depolarizing current (30 ms; 5 pA increments). **H** The analyses of the rheobase (unpaired t test, t = 0.4723, *p* = 0.6440, *n* = 8 neurons per group, mice = 3 per group). **I** The analyses of the RMP (unpaired t test, t = 0.2851, *p* = 0.7798, *n* = 8 neurons per group, mice = 3 per group). ***p* < 0.01.
